# Identification and characterization of the biosynthetic gene cluster of polyoxypeptin A, a potent apoptosis inducer

**DOI:** 10.1186/1471-2180-14-30

**Published:** 2014-02-08

**Authors:** Yanhua Du, Yemin Wang, Tingting Huang, Meifeng Tao, Zixin Deng, Shuangjun Lin

**Affiliations:** 1 State Key Laboratory of Microbial Metabolism, School of Life Sciences and Biotechnology, Shanghai Jiao Tong University, 800 Dongchuan Road, Shanghai 200240, China; 2 Current address: Department of Chemistry at the Scripps Research Institute, Jupiter, FL 33458, USA

**Keywords:** Biosynthesis, Polyoxypeptin A, Polyketide, Nonribosomal peptide, Apoptosis-inducing activity

## Abstract

**Background:**

Polyoxypeptin A was isolated from a culture broth of *Streptomyces* sp. MK498-98 F14, which has a potent apoptosis-inducing activity towards human pancreatic carcinoma AsPC-1 cells. Structurally, polyoxypeptin A is composed of a C_15_ acyl side chain and a nineteen-membered cyclodepsipeptide core that consists of six unusual nonproteinogenic amino acid residues (N-hydroxyvaline, 3-hydroxy-3-methylproline, 5-hydroxypiperazic acid, N-hydroxyalanine, piperazic acid, and 3-hydroxyleucine) at high oxidation states.

**Results:**

A gene cluster containing 37 open reading frames (ORFs) has been sequenced and analyzed for the biosynthesis of polyoxypeptin A. We constructed 12 specific gene inactivation mutants, most of which abolished the production of polyoxypeptin A and only Δ*plyM* mutant accumulated a dehydroxylated analogue polyoxypeptin B. Based on bioinformatics analysis and genetic data, we proposed the biosynthetic pathway of polyoxypeptin A and biosynthetic models of six unusual amino acid building blocks and a PKS extender unit.

**Conclusions:**

The identified gene cluster and proposed pathway for the biosynthesis of polyoxypeptin A will pave a way to understand the biosynthetic mechanism of the azinothricin family natural products and provide opportunities to apply combinatorial biosynthesis strategy to create more useful compounds.

## Background

**Figure 1:**
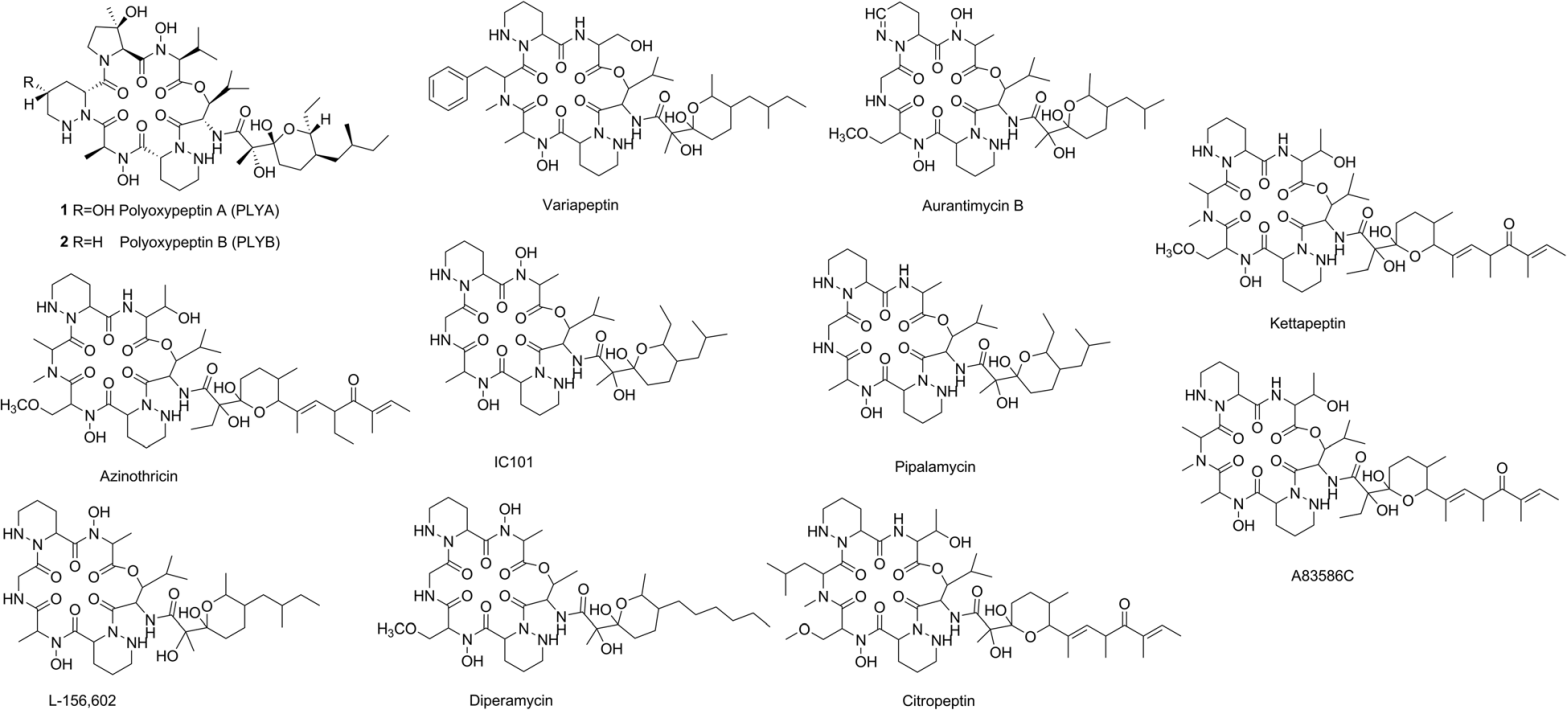
Structures of polyoxypeptin A and B, and other natural products of Azinothricin family.

Polyoxypeptin A (PLYA) was isolated from the culture broth of *Streptomyces* sp. MK498-98 F14, along with a deoxy derivative named as polyoxypeptin B (PLYB), as a result of screening microbial culture extracts for apoptosis inducer of the human pancreatic adenocarcinoma AsPC-1 cells that are highly apoptosis-resistant [1,2]. PLYA is composed of an acyl side chain and a cyclic hexadepsipeptide core that features two piperazic acid units (Figure 1). Structurally similar compounds have been identified from actinomycetes including A83586C [3], aurantimycins [4], azinothricin [5], citropeptin [6], diperamycin [7], kettapeptin [8], IC101 [9], L-156,602 [10], pipalamycin [11], and variapeptin [12] (Figure 1). This group of secondary metabolites was named ‘azinothricin family’ after the identification of azinothricin as the first member in 1986 from *Streptomyces* sp. X-1950.

The compounds in this family exhibit diverse biological activities, such as potent antibacterial, antitumor [13,14], and anti-inflammatory activities [15], and acceleration of wound healing [16]. Both PLYA and PLYB were confirmed to be potent inducers of apoptosis. They can inhibit the proliferation of apoptosis-resistant AsPC-1 cells with IC_50_ values of 0.062 and 0.015 μg/mL. They can also induce early cell death in human pancreatic adenocarcinoma AsPC-1 cell lines with ED_50_ values of 0.08 and 0.17 μg/mL, more efficiently than adriamycin and vinblastine that can’t induce death of AsPC-1 cells even at 30 μg/mL [2]. In addition, they are able to induce apoptotic morphology and internucleosomal DNA fragmentation in AsPC-1 cell lines at low concentrations [17].

Polyoxypeptins (A and B) possess a variety of attractive biosynthetic features in their structures. The C_15_ acyl side chain may present a unique extension unit in polyketide synthase (PKS) assembly line probably derived from isoleucine [18]. The cyclo-depsipeptide core consists of six unusual amino acid residues at high oxidation states, including 3-hydroxyleucine, piperazic acid, N-hydroxyalanine, 5-hydroxypiperazic acid (for PLYA) or piperazic acid (for PLYB), 3-hydroxy - 3-methylproline, and N-hydroxyvaline. The most intriguing is the hydroxylation at α-amino groups of the l-alanine and l-valine, different from that at terminal amino group of ornithine or lysine in siderophore biosynthesis [19]. It is worth to note that (*2S*, *3R*) -3-hydroxy - 3-methylproline presents a synthetic challenge [20]. Both structural novelty and biological activity of polyoxypeptins have spurred much interest in understanding the biosynthetic mechanism and employing biosynthesis and combinatorial biosynthesis to create new polyoxypeptin derives.

Here, we report the identification and characterization of the biosynthetic gene cluster for PLYA based on the genome sequencing, bioinformatics analysis, and systematic gene disruptions. The five stand-alone nonribosomal peptide synthetase (NRPS) domains were confirmed to be essential for PLYA biosynthesis, putatively involved in the biosynthesis of the unusual building blocks for assembly of the peptide backbone. Furthermore, three hydroxylases and two P450 enzymes were genetically characterized to be involved in the biosynthesis of PLYA. Among them, the P450 enzyme PlyM may play a role in transforming PLYB to PLYA.

## Results and discussion

### Identification and analysis of the ply gene cluster

Whole genome sequencing of *Streptomyces* sp. MK498-98 F14 using the 454 sequencing technology yielded 11,068,848 bp DNA sequence spanning 528 contigs. Based on the structural analysis of PLYs, we hypothesized that PLYs are assembled by a hybrid PKS/NRPS system. Bioinformatics analysis of the whole genome revealed at least 20 NRPS genes and 70 PKS genes. Among them, the contig00355 (48439 bp DNA sequence) attracted our attention because it contains 7 putative NRPS genes and 4 PKS genes encoding total 4 PKS modules that perfectly match the assembly of the C_15_ acyl side chain based on the colinearity hypothesis [21]. Moreover, *orf14777* (*plyP*) annotated as an l-proline-3-hydroxylase may be involved in the hydroxylation of 3-methylproline, one of the proposed precursor of PLYA [18]. NRPS analysis program revealed that 7 NRPS genes encode a free-standing peptidyl carrier protein (PCP) (PlyQ), 3 stand-alone thioesterase (TE) domains (PlyI, PlyS, and PlyY), and 3 NRPS modules that are not sufficient for assembly of the hexapeptide. Therefore, we continued to find another relevant contig00067 (83207 bp DNA sequence) contains 4 NRPS genes encoding a free-standing adenylation (A) domain (PlyC) and PCP (PlyD), and 3 NRPS modules. Taken together, the total 6 NRPS modules and 4 PKS modules are sufficient for the assembly of PLYs.

**Figure 2:**
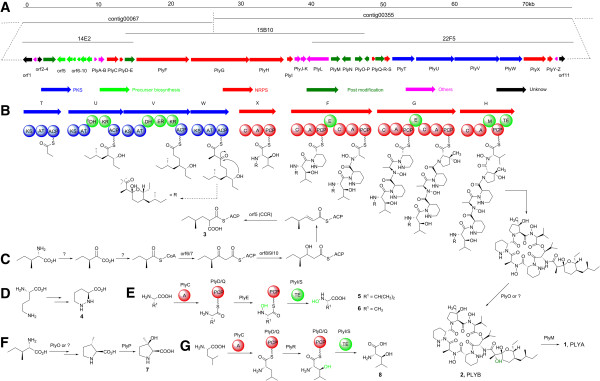
**The biosynthetic gene cluter and proposed biosynthetic pathways for PLYA. A**, Organization of the genes for the biosynthesis of PLYA. Their putative functions were indicated by color-labeling. **B**, the proposed model for PLYA skeleton assembly driven by the hybrid PKS/NRPS system. KS: Ketosynthase; AT: Acyltransferase; ACP: Acyl carrier protein; DH: Dehydratase; KR: Ketoreductase; ER: Enoyl reductase; A: Adenylation domain; PCP: Peptidyl carrier protein; C: Condensation domain; E: Epimerase domain; M: Methyltransferase; TE: Thioesterase. **C**, the proposed pathway for the biosynthesis of **3** (2-(2-methylbutyl)malonyl-ACP). **D**, the biosynthesis of **4** (l-piperazic acid). **E**, the proposed pathway for the biosynthesis of the building blocks **5** (N-hydroxylvaline) and **6** (N-hydroxylalanine). **F** and **G**, the proposed biosynthetic pathways of the building blocks **7** ((*R*)-3-hydroxy-3-methyproline) and **8** (3-hydroxyleucine).

**Figure 3:**
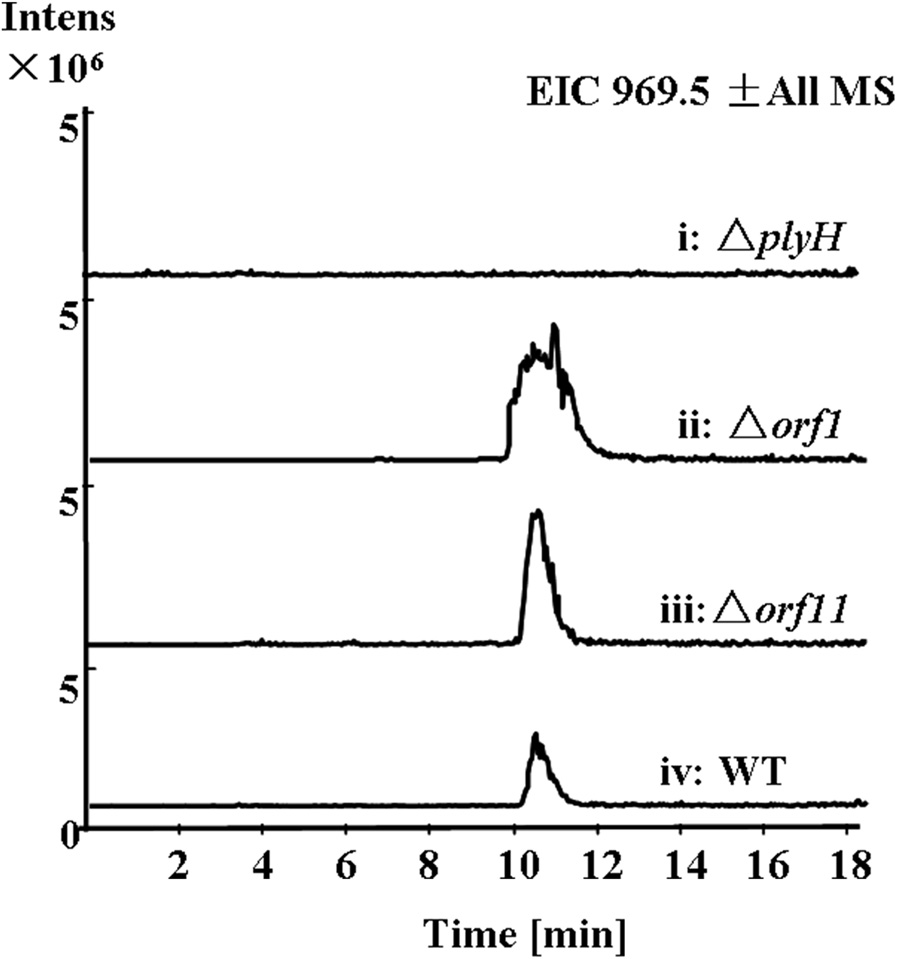
**Verification of the ply gene cluster.** LC-MS analysis (extracted ion chromatograms of m/z [M + H]^+^ 969.5 corresponding to PLYA) of *Streptomyces* sp. MK498-98 F14 wild type (indicated with WT) and mutants (Δ*orf1*, Δ*orf11*, and Δ*plyH*).

To confirm involvement of the genes in these two contigs by disruption of specific NRPS genes, a genomic library of *Streptomyces* sp. MK498-98 F14 was constructed using SuperCos1 [22] and ~3000 clones were obtained. Two pairs of primers (Additional file 1: Table S3) were designed on the base of two hydroxylases (PlyE and PlyP) from the contig00067 and contig00355, respectively, and used to screen the cosmid library using PCR method [23]. 10 positive cosmids derived from the primer of *plyE* and 11 positive cosmids derived from the primer of *plyP* were obtained. Interestingly, these two sets of cosmids overlapped one same cosmid, 15B10, which gave the further evidence that these two contigs belong to the same contig (Figure 2A). Thus, we used 15B10 as a template to fill the gap between these two contigs by PCR sequencing and got a 131,646 bp contiguous DNA sequence (Figure 2A). Subsequently, a NRPS gene *orf14800* (*plyH*) was inactivated by replacement of *plyH* with apramycin resistant gene (*aac(3)IV-oriT*) cassette in the genome of *Streptomyces* sp. MK498-98 F14 (Additional file 1: Scheme S1). The resulting double-crossover mutant completely abolished the production of PLYA (Figure 3, trace i), confirming that the genes in this region are responsible for biosynthesis of PLYs.

Bioinformatics analysis suggested that 37 open reading frames (ORFs, Figure 2A and Table 1) spanning 75 kb in this region were proposed to constitute the *ply* gene cluster based on the functional assignment of the deduced gene products. Among them, 4 modular type I PKS genes (*plyTUVW*) and 4 modular NRPS genes (*plyXFGH*) encoding 4 PKS modules and 6 NRPS modules are present for the assembly of the PLY core structure (Figure 2B). Other 6 NRPS genes (*plyCDQISY*) encode an A domain, two PCPs, and three TEs that are free-standing from the modular NRPSs. They are suggested to be involved in the biosynthesis of nonproteinogenic amino acid building blocks. 6 genes (*orf5-orf10*) are proposed to be involved in the biosynthesis of a novel extender unit for PKS assembly (Figure 2C). There are 6 genes (*orf4* and *plyEMOPR*) encoding putative hydroxylases or oxygenases that are proposed to responsible for the biosynthesis of unusual building blocks or post-modifications (Figure 2D-G). There are 2 ABC transporter genes (*plyJ* and *plyK*) and 4 putative regulatory genes (*orf2*, *plyB*, *plyL*, and *plyZ*). In addition, an aminotransferase gene (*plyN*) is located in the center of the *ply* gene cluster that is probably involved in the biosynthesis of the novel PKS extender unit (**3**) (Figure 2C).

**Table 1:** Deduced functions of ORFs in the biosynthetic gene cluster of PLYA

**Gene**	**Size**^**a**^	**Accession no.**	**Proposed function**	**Homologous protein species**	**Identity/Similarity**
*orf03399*	384	YP_003099796	Nucleotidyl transferase	Actinosynnema mirum DSM 43827	64/73
*orf03396*	309	YP_004903951	putative sugar kinase	Kitasatospora setae KM-6054	50/62
*orf1*	422	YP_003099794	3-dehydroquinate synthase	Actinosynnema mirum DSM 43827	56/69
*orf2*	128	EID72461	MarR family transcriptional regulator	Rhodococcus imtechensis RKJ300	71/83
*orf3*	146	ZP_09957194	Hypothetical protein	Streptomyces chartreusis NRRL 12338	75/84
*orf4*	566	CAJ61212	Putative polyketide oxygenase/hydroxylase	Frankia alni ACN14a	77/83
*orf5*	377	ZP_04706918	Alcohol dehydrogenase BadC	Streptomyces roseosporus NRRL 11379	76/86
*orf6*	312	ZP_06582592	3-oxoacyl-[acyl-carrier-protein] synthase III	Streptomyces roseosporus NRRL 15998	71/82
*orf7*	82	ZP_04706920	Hypothetical protein	Streptomyces roseosporus NRRL 11379	59/75
*orf8*	82	ZP_04706921	Dihydrolipoamide succinyltransferase	Streptomyces roseosporus NRRL 11379	65/81
*orf9*	326	ZP_06582595	2-oxoisovalerate dehydrogenase	Streptomyces roseosporus NRRL 15998	75/87
*orf10*	303	ZP_04706923	Pyruvate dehydrogenase	Streptomyces roseosporus NRRL 11379	74/84
*plyA*	71	YP_640626	MbtH-like protein	Mycobacterium sp. MCS	80/87
*plyB*	225	YP_712760	Putative regulator	Frankia alni ACN14a	76/84
*plyC*	528	YP_712761	A	Frankia alni ACN14a	77/85
*plyD*	77	YP_712762	PCP	Frankia alni ACN14a	85/94
*plyE*	395	YP_712763	Putative hydroxylase	Frankia alni ACN14a	76/86
*plyF*	2583	ABV56588	C-A-PCP-E-C-A-PCP	Kutzneria sp. 744	56/68
*plyG*	2809	ZP_05519638	C-A-PCP-E-C-A-PCP	Streptomyces hygroscopicus ATCC 53653	73/82
*plyH*	1662	BAH04161	C-A-M-PCP-TE	Streptomyces triostinicus	72/82
*plyI*	247	YP_712767	TE	Frankia alni ACN14a	80/87
*plyJ*	312	YP_003112824	Daunorubicin resistance ABC transporter	Catenulispora acidiphila DSM 44928	78/90
*plyK*	253	YP_712769	ABC transporter system	Frankia alni ACN14a	71/81
*plyL*	1043	YP_003112826	Transcriptional regulator	Catenulispora acidiphila DSM 44928	72/80
*plyM*	412	AAT45271	Cytochrome P450 monooxygenase	Streptomyces tubercidicus	43/59
*plyN*	450	ZP_04604097	Aminotransferase class I and II	Micromonospora sp. ATCC 39149	58/70
*plyO*	308	ZP_03862696	Polyketide fumonisin	Kribbella flavida DSM 17836	48/64
*plyP*	270	ZP_04292518	L-proline 3-hydroxylase type II	Bacillus cereus R309803	32/51
*plyQ*	88	YP_712776	PCP	Frankia alni ACN14a	76/89
*plyR*	415	YP_712777	Cytochrome P450 monooxygenase	Frankia alni ACN14a	77/85
*plyS*	245	AAT45287	TE	Streptomyces tubercidicus	70/81
*plyT*	1031	YP_712779	KS-AT-ACP	Frankia alni ACN14a	71/80
*plyU*	1872	YP_712780	KS-AT-DH-KR-ACP	Frankia alni ACN14a	69/79
*plyV*	2199	YP_712781	KS-AT-DH-ER-KR-ACP	Frankia alni ACN14a	70/78
*plyW*	1041	YP_712782	KS-AT-ACP	Frankia alni ACN14a	72/82
*plyX*	1080	YP_712783	C-A-PCP	Frankia alni ACN14a	62/72
*plyY*	253	ZP_04472110	TE	Streptosporangium roseum DSM 43021	70/78
*plyZ*	79	YP_001612061	LysR family transcriptional regulator	Sorangium cellulosum	65/77
*orf11*	287	YP_702564	Hypothetical protein	Rhodococcus jostii RHA1	56/73
*orf14742*	170	YP_002777514	Hypothetical protein ROP_03220	Rhodococcus opacus B4	51/63

^a^Numbers are in amino acids.

Upstream of the *ply* gene cluster, three genes, *orf03394* (*orf1*), *orf03396* and *orf03399*, encoding proteins with similarities to 3-dehydroquinate synthase, sugar kinase and nucleotidyl transferase respectively, seemingly have no relationship with the biosynthesis of PLYA. *orf03392* (*orf2*), adjacent to *orf1*, is predicted to encode a protein with similarity to a transcriptional regulator, which may be involved in the biosynthesis of PLYs*.* Downstream of the *ply* gene cluster, three genes, *orf14746* (*plyZ*), *orf14744* (*orf11*) and *orf14742* encode proteins with similarities to LysR family transcriptional regulator, hypothetical protein ROP_29250 and hypothetical protein ROP_03220. To prove that the genes beyond this cluster are not related to PLY biosynthesis, we inactivated *orf1* and *orf11*. The resulting mutants have no effect on the PLYA production (Figure 3, trace ii and iii), indicating that the 37 ORFs-contained *ply* gene cluster is responsible for the PLYs biosynthesis.

### Assembly of the C_15_ acyl side chain by PKSs

Within the *ply* cluster, 4 modular type I PKS genes (*plyTUVW*) encode four PKS modules, the organization of which is accordant with the assembly of the C_15_ acyl side chain of PLYA via three steps of elongation from the propionate starter unit (Figure 2B). Both PlyT and PlyW consist of ketosynthase (KS), acyltransferase (AT), and acyl carrier protein (ACP). However, the active site Cys (for transthioesterification) of the PlyT-KS is replaced with Gln (Additional file 1: Figure S1), so it belongs to the so called “KS_Q_” that often occurs in the loading module of PKS system [24]. Therefore, PlyT acts as a loading module for formation of the propionate starter unit by catalyzing decarboxylation of methylmalonyl group after tethering onto ACP (Figure 2B). The conserved regions of AT domain including the active site motif GHSQG [25] in both PlyT and PlyW (Additional file 1: Figure S2), along with substrate specificity code (YASH) [26] indicate that both ATs are specific for methylmalonyl-CoA, consistent with the structure of the side chain of PLYA (Figure 2B). In PlyU, in addition to KS, AT, and ACP domains, a dehydratase (DH) domain and a ketoreductase (KR) domain are present. However, the DH domain here is believed to be nonfunctional because the key amino acid residue H of the conserved motif HxxxGxxxxP [27] is replaced by Gln (Additional file 1: Figure S3). The conserved motif of PlyU-AT for substrate selectivity is VPGH, neither including the serine residue in YASH for methylmalonyl-CoA nor phenylalanine residue in HAFH for malonyl-CoA (Additional file 1: Figure S2). These changes may broaden the substrate binding pocket and enhance hydrophobicity of the substrate binding pocket, supporting that PlyU is able to recognize 2-(2-methylbutyl)malonyl 3 as an unusual extender unit (Figure 2C). Compared to PlyU, PlyV contains an active DH domain and an enoyl reductase (ER) domain. The conserved motif (HAFH) of PlyV-AT signifies it specific for malonyl-CoA as the extender unit (Figure 2B and Additional file 1: Figure S2). Taken together, PlyTUVW seem to be sufficient for the assembly of the C_15_ acyl side chain of PLYA.

### Biosynthesis of 2-(2-methylbutyl)malonyl extender unit 3

**Figure 4:**
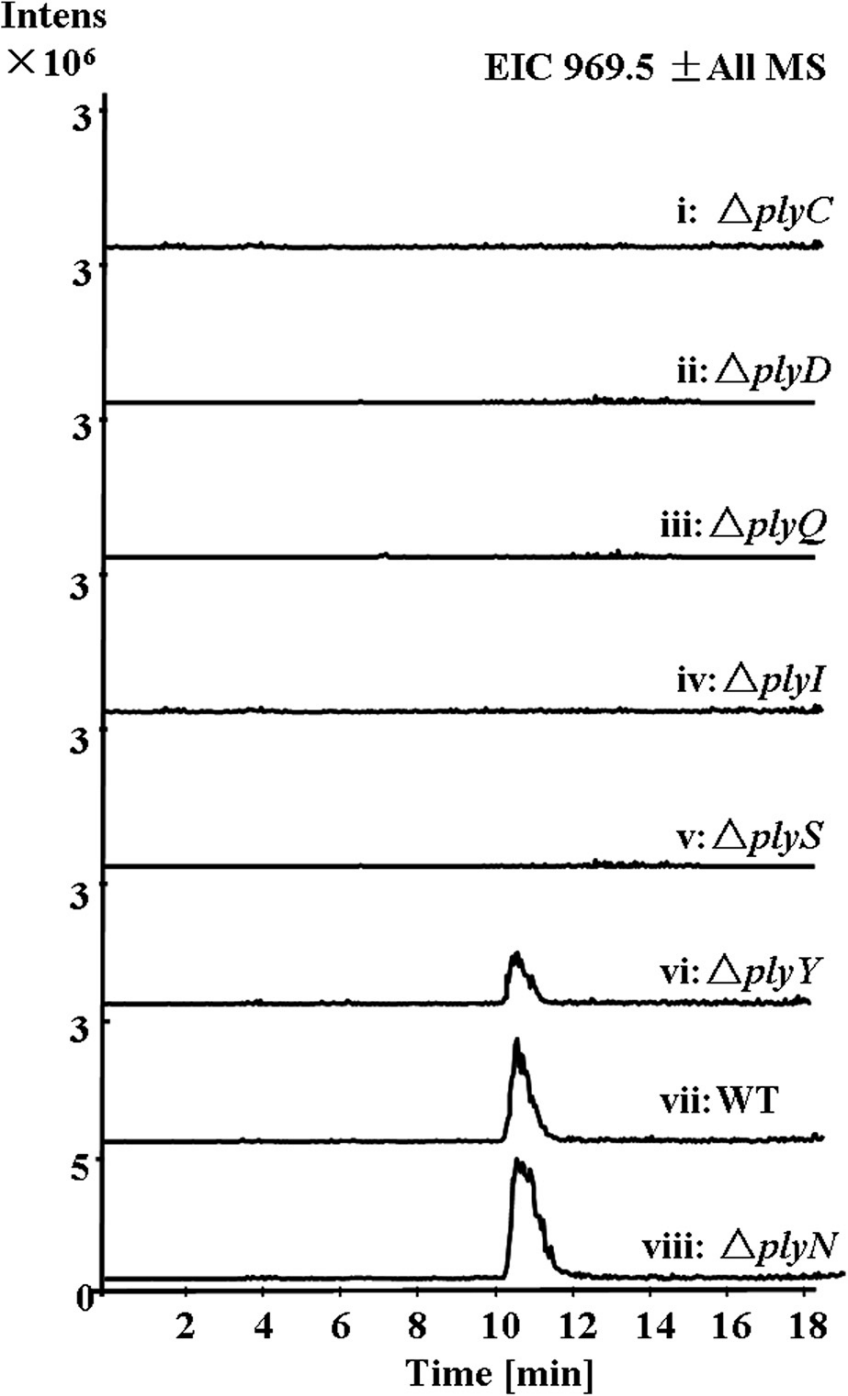
**Characterization of the discrete NRPS domains and aminotransferase in vivo.** LC-MS analysis (extracted ion chromatograms of m/z [M + H]^+^ 969.5 corresponding to PLYA) of *Streptomyces* sp. MK498-98F14 wild type (WT) and mutants (Δ*plyC*, Δ*plyD*, Δ*plyQ*, Δ*plyI,* Δ*plyS*, Δ*plyY* and Δ*plyN*).

The structural analysis of PLYs and PKS architecture suggest that an unusual PKS extender unit 2-(2-methylbutyl)malonyl-CoA (or ACP, **3**) is required for the assembly of the C_15_ acyl side chain of PLYs. The biosynthesis of the 2-(2-methylbutyl)malonyl-CoA (or ACP) extender unit **3** would involve a reductive carboxylation mediated by a crotonyl-CoA reductase/carboxylase (CCR) homolog. Similar reactions have been reported for formation of ethylmalony-CoA [28,29], 2-(2-chloroethyl)malonyl-CoA [30], and hexylmalonyl-CoA [31], as well as proposed for involvement of biosynthesis of cinnabaramides [32], thuggacins [33], sanglifehrins [34], germicidins and divergolides [35], ansalactams [36] and many other natural products. Analysis of the *ply* cluster reveals *orf5* encoding a CCR TgaD homolog (identity/similarity, 46%/59%) that was proposed to be involved in the biosynthesis of hexylmalonyl-CoA, an extender unit for the assembly of thuggacin [33]. *orf6*, adjacent to *orf5*, encodes a protein shared 71% identity and 81% similarity with 3-oxoacyl-ACP synthase III from *S. roseosporus* NRRL 15998. The gene *orf7*, located upstream of *orf6*, encodes an ACP that contains a catalytic motif DLDLDSL (the Serine is for phosphopantethein modification) [24]. The presence of these two genes indicates that the extender unit 2-(2-methylbutyl)malonyl may be tethered to ACP, not to CoA. In study of the biosynthesis of isobutylmalonyl-CoA extender unit for germicidins and divergolides, CCR, KSIII and HBDH (a 3-hydroxybutyryl-CoA hydrogenase) are transcribed in the same operon [35]. *orf567* and other three genes *orf8910* also constitute an operon (Figure 2A). The genes *orf8910* encode α-keto acid dehydrogenase E2 component, E1 component β and α subunits, respectively, suggesting their involvement of the biosynthesis of **3** by reduction of the β-keto group (Figure 2C). Given that the previous feeding study with isotope-labeled precursor suggested this 2-(2-methylbutyl)malonyl unit derived from isoleucine via a transamination [18], we proposed that an aminotransferase is required for the formation of α-keto acid, as shown in Figure 2C. *plyN* is the only identified aminotransferase gene, so we constructed the Δ*plyN* mutant by replacement of the *plyN* gene with the *aac(3)IV-oriT* cassette (Additional file 1: Scheme S2). However, Δ*plyN* was found no effect on the PLYA production (Figure 4, trace viii), so we assume that other aminotransferases may mediate this transamination for the incorporation of C_5_ unit of isoleucine into 3 (Figure 2C).

### Assembly of the cyclodepsipeptide by NRPSs

After the C_15_ acyl side chain is assembled by 4 modular PKSs, it is transferred to 3-hydroxyleucine via an amide bond formation catalyzed by a NRPS, thus initiating the assembly of the peptide core. Within the biosynthetic gene cluster, there are 4 genes *plyFGHX* encoding modular NRPS proteins. Both PlyF and PlyG consist of two modules with seven domains (C-A1-PCP-E-C-A2-PCP) (Figure 2B). Active epimerase (E) domains are present indicating that the amino acids activated by PlyF-A1 and PlyG-A1 should be converted into d-configuration. Among the six nonproteinogenic amino acid residues, only two piperazic acid residues are d-configuration, so these two A domains (PlyF-A1 and PlyG-A1) are proposed to recognize and activate l-piperazic acid (**4**, Figure 2D) that was confirmed to be derived from l-ornithine [37]. This assumption can be supported by the findings that PlyF-A1 shares 52-59% identity and 64-69% similarity to PlyG-A1, KtzH-A1 [38], and HmtL-A1 [39] (Additional file 1: Figure S4), and as well as the substrate specificity-conferring ten amino acids (DVFSVASYAK for PlyF-A1 and DVFSIAAYAK for PlyG-A1) are highly analogous to those of KtzH-A1 (DVFSVGPYAK) and HmtL-A1 (DVFSVAAYAK) [40,41]. Both KtzH-A1 and HmtL-A1 were proposed to recognize and activate l-piperazic acid [38,39]. PlyH contains five domains (C-A-M-PCP-TE) with a thioesterase (TE) domain present, indicating that PlyH is the last module of PLY NRPS system and responsible for the release and cyclization of the peptide chain via an ester bond formation. It is striking that an active methyltransferase (M) domain (containing the SAM-binding sites EXGXGXG) is present in the PlyH [42], but no N-methyl group is present in the structure of PLYs. The presence of this M domain remains enigmatic. Based on the PLY structure analysis and NRPS machinery [43], PlyH-A is proposed to recognize N-hydroxyvaline (**5**, Figure 2E) as its substrate, but not valine because its substrate specificity-conferring codon sequences (DAPFEALVEX) are significantly distinct from those found for valine-specificity (DALWMGGTFK) [44]. Subsequently, the whole sequence of PlyH-A shows 76% identity and 83% similarity to that of PlyF-A2, indicating that PlyF-A2 is specific for N-hydroxyalanine (**6**, Figure 2E and Additional file 1: Figure S5). These assignments are consistent with the amino acid sequence of the peptide core of PLYs. Finally, according to the collinearity of the NRPS modules and the building blocks of the NRPS-derived products, PlyG-A2 and PlyX would be proposed to recognize and activate (*R*)-3-hydroxy-3-methylproline (**7**, Figure 2F) and 3-hydroxyleucine (**8**, Figure 2G), respectively, although we can’t predict their substrates based on their substrate specificity codons (Additional file 1: Table S4). Taken together, six NRPS modules activate six non-natural amino acids, and the substrate recognized by each domain is exactly consistent with the structure of the cyclic depsipeptide of PLYs (Figure 2B).

### Biosynthesis of nonproteinogenic amino acid building blocks

Except for the modular NRPSs, there are six discrete NRPS genes present in the *ply* gene cluster (Table 1 and Figure 2A), identified as an A domain (PlyC), two PCP domains (PlyD, PlyQ) and three TE domains (PlyI, PlyS, PlyY). To test whether these six free-standing domains were involved in the biosynthesis of PLYA, we constructed their disruption mutants by gene replacement with the *aac(3)IV-oriT* cassette (Additional file 1: Scheme S3-8). The mutant strains (Δ*plyC*, Δ*plyD*, Δ*plyQ*, Δ*plyI* and Δ*plyS*) completely abolished the production of PLYA (Figure 4, traces i-v), indicating that these 5 discrete NRPS domains are essential for the PLYA biosynthesis. However, the Δ*plyY* mutant strain still produced PLYA, but the productivity decreased in comparison with that of the wild type strain (Figure 4, trace vi and vii). Therefore, PlyY may act as a type II TE, probably playing an editing role in the biosynthesis of PLYA by hydrolyzing misincorporated building blocks. Multiple sequence alignment reveals that PlyY and typical type II TEs contain a conserved motif (GHSXG) and catalytic triad S/C-D-H that is consistent with hydrolytic function (Additional file 1: Figure S6) [45-47]. This catalytic triad is also present in PlyI and PlyS, indicating the hydrolytic function of PlyI and PlyS, as shown by Figure 2E and G.

The discrete NRPS domains have been found in many NRPS assembly lines responsible for the formation of nonproteinogenic building blocks [21,48]. For example, the conversion of proline to pyrrole-2-carboxylic acid, which is a precursor for the biosynthesis of pyoluteorin, prodigiosin, and clorobiocin [49], occurs while proline is activated by a discrete A domain and covalently tethered in a thioester linkage to a T domain. Since all the A domains of six modular NRPSs in the PLY biosynthetic pathway are proposed to recognize and activate nonproteinogenic amino acid building blocks, PlyCDQIS are assumed to be responsible for the formation of several monomers of PLYs from the natural amino acids. Given that we can’t predict the substrate based on the key residues of the substrate-binding pocket of PlyC (A domain), we propose that PlyC may activate multiple amino acids such as alanine and valine or leucine, and tether them to the corresponding PCPs (PlyD and PlyQ). After N-hydroxylation of alanine and valine (Figure 2E) as well as β-hydroxylation of leucine (Figure 2G), the matured building blocks are proposed to be released by discrete TEs (PlyI or PlyS, respectively) and activated again by PlyF-A2, PlyH, and PlyX, respectively (Figure 2B). Such processes are rare events in typical NRPS-driven biosynthetic pathways [21].

**Figure 5:**
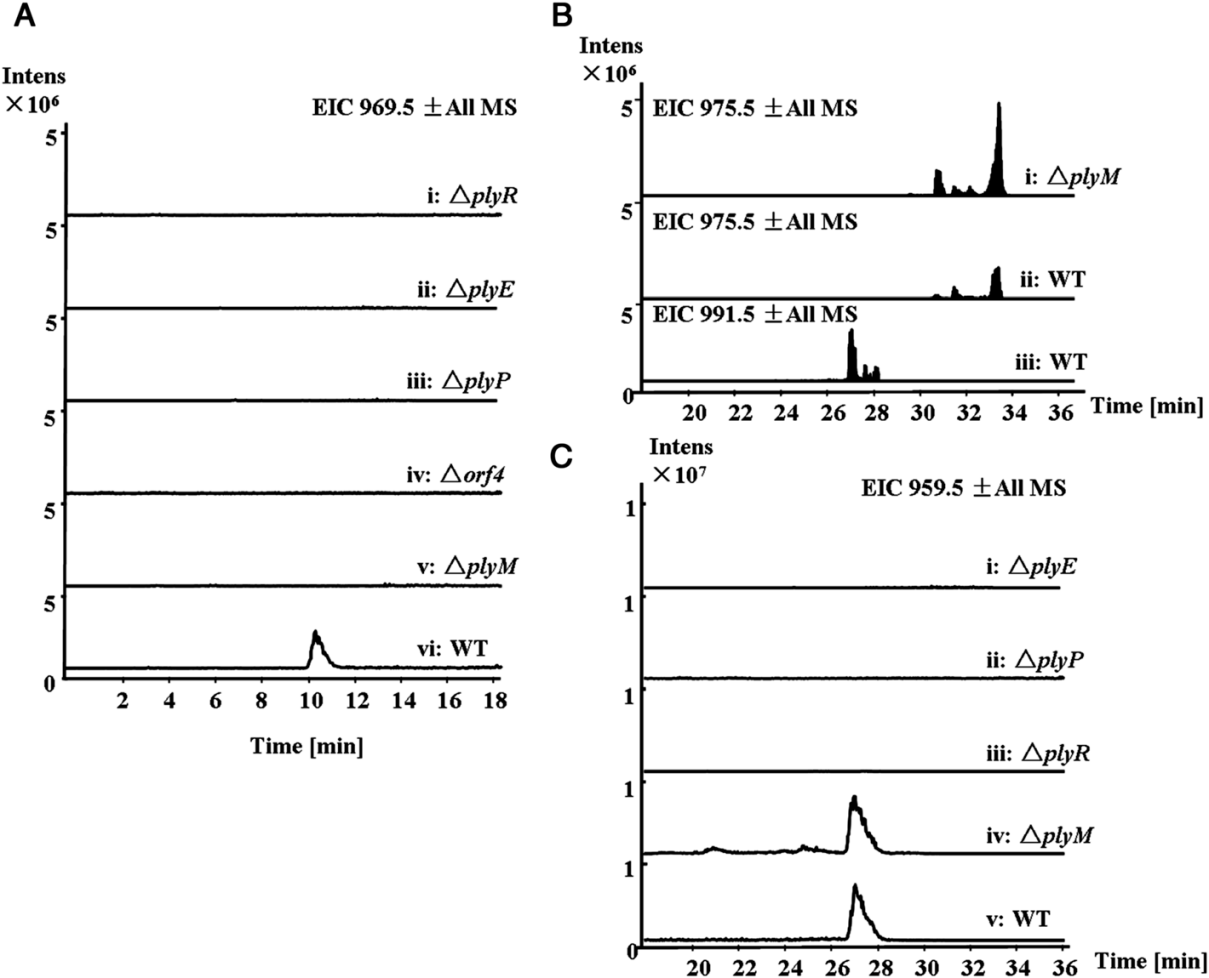
**Characterization of the genes encoding hydroxylases or oxygenases. A**, LC-MS analysis (extracted ion chromatograms of m/z [M + H]^+^ 969.5 corresponding to PLYA) of *Streptomyces* sp. MK498-98F14 wild type (WT) and mutants (Δ*plyE*, Δ*plyP*, Δ*plyR*, Δ*orf4*, and Δ*plyM*). **B**, LC-MS analysis (extracted ion chromatograms of m/z [M + Na]^+^ 975.5 and 991.5 corresponding to PLYB and PLYA) of *Streptomyces* sp. MK498-98F14 wild type (WT) and the Δ*plyM* mutant. **C**, LC-MS analysis (extracted ion chromatograms of m/z [M + Na]^+^ 959.5 corresponding to the putative biosynthetic intermediate of PLYA lacking two hydroxyl groups) of *Streptomyces* sp. MK498-98F14 wild type (WT) and mutants (Δ*plyE*, Δ*plyP*, Δ*plyR* and Δ*plyM*). **B** was performed under the conditions: 35-95% B (linear gradient, 0–20 min), 100% B (21–25 min), 35% B (25-40 min) at the flow rate of 0.3 mL/min.

The depsipeptide core of PLYA is composed of 6 amino acids, 5 of which are hydroxylated. There are 6 genes encoding putative hydroxylases or oxygenases. For example, *plyR* encodes a cytochrome P450 monooxygenase that shows high homology (37% identity and 54% similarity) to NikQ that was demonstrated to catalyze β-hydroxylation of histidine tethered to PCP, so we could propose that PlyR may be involved in the formation of β-hydroxyleucine building block (Figure 2G). Indeed, inactivation of *plyR* resulted in loss of ability to produce PLYA (Figure 5A, trace i). Given that FAD-dependent monooxygenase CchB has been reported to catalyze the N-hydroxylation of the δ-amino group of ornithine in the biosynthetic pathway of the siderophore coelichelin [50], we proposed that PlyE, a FAD-dependent monooxygenase, may be responsible for N-hydroxylation of alanine and valine when they are activated and tethered to a PCP by A domain PlyC (Figure 2E). The Δ*plyE* mutant lost ability to produce PLYA (Figure 5A, trace ii), indicating its possible role in formation of N-hydroxyalanine and N-hydroxyvaline. PlyP, a l-proline 3-hydroxylase, should be responsible for hydroxylation of 3-methyl-l-proline that is biosynthesized from l-isoleucine demonstrated by isotope-feeding study (Figure 2F) [18]. Inactivation of *plyP* indeed abolished the production of PLYA (Figure 5A, trace iii). Recently, Tang and co-workers have reported that an α-ketoglutarate dependent dioxygenase EcdK catalyzes a sequential oxidations of leucine to form the immediate precursor of 4-methylproline [51]. In the *ply* cluster, the only gene *plyO* encodes an α-ketoglutarate dependent dioxygenase, but it doesn’t share any homology to EcdK. In contrast, PlyO shows 48% identity and 64% similarity to phytanoyl-CoA dioxygenase (YP_003381511 from *Kribbella flavida* DSM 17836). It remains unclear whether PlyO may be responsible for the hydroxylation of the carbon adjacent to the acyl group of the C_15_ acyl side chain or for the formation of 3-methyl-l-proline from l-isoleucine. *orf4* encodes a FAD-binding oxygenase or hydroxylase with high homology to type II PKS-assembled aromatic compounds hydroxylase (Table 1). Its role in biosynthesis of PLYA remains unclear, but it might be involved in the biosynthesis of a building block because its inactivation abolished the PLY production (Figure 5A, trace iv).

Piperazic acid is an attractive building block of many complex secondary metabolites such as Antrimycin [52], Chloptosin [53], Himastatin [39], Luzopeptin [54], Quinoxapeptin [55], Lydiamycin [56], Piperazimycin [57] and Sanglifehrin [58]. The detailed biosynthetic mechanisms by which piperazic acid are formed are not well understood. Recently, Walsh and coworkers demonstrated that KtzI, a homolog of lysine and ornithine N-hydroxylases catalyzes the conversion of ornithine into piperazic acid in kutzneride biosynthetic pathway [37]. No such a homolog was found in the *ply* gene cluster, but two putative homologs are located outside the *ply* gene cluster (Orf11257 and Orf14738), suggesting that the biosynthesis of piperazic acid may follow the same pathway (Figure 2D).

### Genes putatively for post-modifications

Most modifications in PLYA biosynthesis take place for the formation of the non-natural building blocks. Recently, Ju and co-workers demonstrated that a cytochrome P450 monooxygenase HtmN catalyzes the hydroxylation of the piperazic acid after peptide formation [59]. There are two cytochrome P450 monooxygenase genes (*plyM* and *plyR*) in the *ply* cluster. PlyR was proposed to hydroxylate leucine that is tethered to a PCP, so we would assume that PlyM may catalyze the hydroxylation of piperazic acid unit as a post-modification although it doesn’t show any homology to HmtN [39]. To test this hypothesis, we constructed the double-crossover mutant by replacement of *plyM* with the *aac(3)IV-oriT* gene cassette that is not producing PLYA (Figure 5A, trace v), only accumulating PLYB (Figure 5B). These findings indicate that PlyM is responsible for the conversion of PLYB into PLYA (Figure 2B). To test whether other oxygenases or hydroxylases are involved in the post-modifications, the mass corresponding to the putative intermediate of PLYA lacking two hydroxyl groups was monitored for the mutant strains (Figure 5C). This mass is only detected from the fermentation broth of wide type and Δ*plyM* strains (Figure 5C, trace v and iv), not from other mutant strains (Δ*plyE*, Δ*plyP* and *ΔplyR*) indicating that the assembly of PLYA and possible intermediates is abolished. These data may support that these genes are involved in the formation of building blocks, not post-modifications. They also indicate that it is very likely to have two steps of post-hydroxylation modifications for maturation of PLYA (Figure 2B). When and how the hydroxylation at the α-carbon of the C_15_ acyl side chain takes place are still unclear.

## Conclusions

We identified and characterized the *ply* gene cluster composed of 37 open reading frames (ORFs) by genomic sequencing and systematic gene disruptions. The biosynthetic pathway has been proposed based on bioinformatics analysis, the structural analysis of PLYs and genetic data. It was demonstrated that five discrete NRPS domains are essential for the biosynthesis of PLYs and proposed their roles in maturation of three unusual amino acid building blocks. The proposed biosynthetic pathway for PLYs will open the door to understand the biosynthesis of this family of secondary metabolites and set a stage to explore combinatorial biosynthesis to create new compounds with improved pharmaceutical properties.

### Ethics statement

This study doesn’t involve human subjects or materials.

## Methods

### Strains, plasmids, primers and culture conditions

Strains, plasmids and primers used in the study are summarized in Additional file 1: Tables S1, S2 and S3 of the supplemental material. *Escherichia coli* strains were cultured on Luria-Bertani (LB) broth and agar medium at 37°C. *Streptomyces* sp. MK498-98 F14 and its mutant strains were cultivated at 30°C on the medium (yeast extract 0.4%, glucose 0.4%, malt extract 1%, agar 1.2%, pH 7.2) for sporulation and on 2CM [60] medium (soluble starch 1%, tryptone 0.2%, NaCl 0.1%, (NH_4_)_2_SO_4_ 0.2%, K_2_HPO_4_ 0.1%, MgSO_4_ 0.1%, CaCO_3_ 0.2%, agar 1.2% with 1 mL inorganic salt solution per liter, pH7.2) for conjugation. For fermentation, mycelia of strain MK498-98 F14 and its mutants from the solid plates were inoculated into a 500-mL Erlenmeyer flask containing 100 mL of a medium composed of glucose 1%, potato starch 1%, glycerol 1%, polypepton 0.5%, meat extract 0.5%, sodium chloride 0.5%, and calcium carbonate 0.32% (adjusted to pH 7.4) [2]. The culture was incubated at 28°C for six days on a rotatory shaker at 220 rpm.

### General genetic manipulations and reagents

The general genetic manipulation in *E. coli* and *Streptomyces* were carried out following the standard protocols [22]. PCR amplifications were performed on a Veriti thermal cycler (Applied Biosystems, Carlsbad, CA) using Taq DNA polymerase. DNA fragments and PCR products were purified from agarose gels using a DNA Gel Extraction Kit (Omega). Primers were synthesized in Sangong Biotech Co. Ltd. Company (Shanghai, China). All DNA sequencing was accomplished at Shanghai Majorbio Biotech Co. Ltd (Shanghai, China). Restriction enzymes were purchased from New England Biolabs (Ipswich, MA) and Fermentas (St. Leon-Rot, Germany). Taq DNA polymerase and DNA ligase were purchased from Takara Co. Ltd. Company (Dalian, China).

### Genomic library construction and screening

A genomic cosmid library of *Streptomyces* sp. MK498-98 F14 derived from SuperCos1 was constructed according to the procedure as described by the SuperCos1 Cosmid Vector Kit. *E. coli* EPI300™-T1^R^, instead of *E.coli* XL1-Blue MR, was used as the host strain. The total number of recombinant clones was about 3000 and then stored at −70°C. Two pairs of primers for two hydroxylase genes, *orf0337*4 (*plyE*) and *orf14777* (*plyP*) were designed and used to screen the genomic cosmid library by PCR.

### Genome sequencing and analysis

Genome sequencing was accomplished by 454 sequencing technology. Open reading frames were analyzed using the Frame Plot 3.0 beta online [61], and the analysis of the deduced function of the proteins were carried out by the NCBI website [62]. Primer design, multiple nucleotide sequence alignments and analysis were performed using the BioEdit. The NRPS-PKS architecture was analyzed by NRPS-PKS online website (http://nrps.igs.umaryland.edu/nrps/) [63] and the prediction of ten amino acid of the conserved substrate-binding pocket of the A domain was performed using the online program NRPS predictor (http://ab.inf.unituebingen.de/toolbox/index.php?view=domainpred) [64].

### Construction of gene inactivation mutants

All the mutant strains in this study were generated by homologous recombination according to the standard method [65]. The target genes were replaced with an apramycin-resistance gene from pIJ773 on SuperCos1 by traditional PCR-targeting technique. Then the recombinant plasmids were transformed into *E. coli* S17-1 cells for conjugation. The exconjugants would appear three days later and could be transferred to a new growth medium supplemented with apramycin (60 μg/mL) and nalidixic acid (100 μg/mL). Double-crossover mutants were identified through diagnostic PCR with corresponding primers (Additional file 1: Table S3).

### LC-MS analyses of wild type and mutant strains

After finishing the fermentation, the culture broth of wild type and mutant strains were extracted by equal volume of ethyl acetate. The supernatant of the ethyl acetate phase was concentrated by rotary evaporator under the reduced pressure and finally dissolved in methanol (400 μL) for the LC-MS analysis using the Agilent 1100 series LC/MSD Trap system. The conditions for the LC-MS analysis are as follows: 55-100% B (linear gradient, 0–25 min, solvent A is water containing 0.1% formic acid, solvent B is acetonitrile containing 0.1% formic acid), 100% B (26–30 min) at the flow rate of 0.3 mL/min with a reverse-phase column ZORBAX SB-C18 (Agilent, 5 μm, 150 mm × 4.6 mm). Figure 4B was recorded with the conditions: 35-95% B (linear gradient, 0–20 min), 100% B (21–25 min), 35%B (25–40 min) at the flow rate of 0.3 mL/min.

### Nucleotide sequence accession number

The sequence of the polyoxypeptin A biosynthetic gene cluster was deposited in GenBank with accession number KF386858.

## Competing interests

The authors declare that they have no competing interests.

## Authors’ contributions

SL designed this study; YD, YW, TH performed the experiments; YD, MT, ZD and SL analyzed data; YD and SL wrote this manuscript; MT and ZD edited this manuscript; All authors read and approved the final manuscript.

## Supplementary Material

Additional file 1 **Electronic supplementary materials are available: bacterial strains (Table S1), plasmids (Table S2), primers (Table S3), and the substrate-specific codon sequences (Table S4); sequence alignment (Figures S1-6) and mutant construction (Schemes S1-8).** Click here for file
