# Supplementary material for: Identification and characterization of the biosynthetic gene cluster of polyoxypeptin A, a potent apoptosis inducer
Source: BMC Microbiol. 2014 Feb 8;14:30. doi: 10.1186/1471-2180-14-30 (PMC3943440; doi:10.1186/1471-2180-14-30)
Supplement: Additional file 1 — Electronic supplementary materials are available: bacterial strains (Table S1), plasmids (Table S2), primers (Table S3), and the substrate-specific codon sequences (Table S4); sequence alignment (Figures S1-6) and mutant construction (Schemes S1-8). [file 1471-2180-14-30-S1.pdf]

Supplementary materials

Identification and Characterization of the Biosynthetic Gene Cluster of Polyoxypeptin A, a Potent Apoptosis Inducer

Yanhua Du[a], Yemin Wang[a], Tingting Huang[b], Meifeng Tao[a], Zixin Deng[a], Shuangjun Lin*[a]

[a] State Key Laboratory of Microbial Metabolism, School of Life Sciences and Biotechnology, Shanghai Jiao Tong University, 800 Dongchuan Road, Shanghai 200240, China. Email:linsj@sjtu.edu.cn

[b] Current address: Department of Chemistry at The Scripps Research Institute, Jupiter, FL 33458

Contents of supplementary materials Pages

**1 Tables**	Table S1. Strains used in this study	2
	Table S2. Plasmids used in this study	3
	Table S3. Primers used in this study	4-6
	Table S4. The substrate specificity codons of seven A domain in this study	7
**2 Figures**	Figure S1. Alignment of four KS domains of the *ply* gene cluster	8
	Figure S2 Alignment of four AT domains	9
	Figure S3 Alignment of DH domains	10
	Figure S4 Alignment of A domains activating piperazic acid	11
	Figure S5 Alignment of PlyF-A2 and PlyH-A.	12
	Figure S6 Alignment of Type I and Type II TE domains	13
**3. Schemes**	Scheme S1. Construction and the genotype verification of the Δ*plyH* mutant DYH1	14
	Scheme S2. Construction and the genotype verification of the Δ*plyN* mutant DYH11	15
	Scheme S3. Construction and the genotype verification of the Δ*plyC* mutant DYH2	16
	Scheme S4 Construction and the genotype verification of the Δ*plyD* mutant DYH3.	17
	Scheme S5. Construction and the genotype verification of the Δ*plyQ* mutant DYH4	18
	Scheme S6. Construction and the genotype verification of the Δ*plyI* mutant DYH5.	19
	Scheme S7. Construction and the genotype verification of the Δ*plyS* mutant DYH6.	20
	Scheme S8. Construction and the genotype verification of the Δ*plyY* mutant DYH7	21
**4. reference**		22

1. Supplementary Tables

Table S1. Strains used in this study

**Strain**	**Characteristic(s)**	**Source/Reference**
**Streptomyces**		
Streptomyces sp. MK498-98F14	Wild-type producer for Polyoxypeptin A	(1)
DYH1	*PlyH* mutant, Apr^R	This work
DYH2	*PlyC* mutant, Apr^R	This work
DYH3	*PlyD* mutant, Apr^R	This work
DYH4	*PlyQ* mutant, Apr^R	This work
DYH5	*PlyI* mutant, Apr^R	This work
DYH6	*PlyS* mutant, Apr^R	This work
DYH7	*PlyY* mutant, Apr^R	This work
DYH8	*PlyE* mutant, Apr^R	This work
DYH9	*PlyM* mutant, Apr^R	This work
DYH10	*Orf11* mutant, Apr^R	This work
DYH11	*PlyN* mutant, Apr^R	This work
DYH12	*PlyP* mutant, Apr^R	This work
DYH13	*Orf4* mutant, Apr^R	This work
DYH14	*PlyR* mutant, Apr^R	This work
DYH15	*Orf1* mutant, Apr^R	This work
E.coli		
EPI300™-T1^R	Host for genomic library construction	Stratagene
DH10B	Host for general cloning	Invitrogen
S17-1	*E.coli* general cloning and conjugation donor strain	(2)

Table S2. Plasmids used in this study

**Plasmids**	**Characteristic(s)**	**Source/Reference**
Supercos 1	Cosmid vector for genomic library construction	Stratagene
pIJ773	*aac(3)IV*	PCR-targeting protocol
pDYH1	Cosmid 15B10 with *PlyH* replaced by *aac(3)IV-oriT* cassette	This work
pDYH2	Cosmid 29G1 with *PlyC* replaced by *aac(3)IV-oriT* cassette	This work
pDYH3	Cosmid 29G1 with *PlyD* replaced by *aac(3)IV-oriT* cassette	This work
pDYH4	Cosmid 27D11 with *PlyQ* replaced by *aac(3)IV-oriT* cassette	This work
pDYH5	Cosmid 15B10 with *PlyI* replaced by *aac(3)IV-oriT* cassette	This work
pDYH6	Cosmid 27D11 with *PlyS* replaced by *aac(3)IV-oriT* cassette	This work
pDYH7	Cosmid 22F5 with *PlyY* replaced by *aac(3)IV-oriT* cassette	This work
pDYH8	Cosmid 29G1with *PlyE* replaced by *aac(3)IV-oriT* cassette	This work
pDYH9	Cosmid 8D6 with *PlyM* replaced by *aac(3)IV-oriT* cassette	This work
pDYH10	Cosmid 22F5with *orf11* replaced by *aac(3)IV-oriT* cassette	This work
pDYH11	Cosmid 8D6with *PlyN* replaced by *aac(3)IV-oriT* cassette	This work
pDYH12	Cosmid 27D11with *PlyP* replaced by *aac(3)IV-oriT* cassette	This work
pDYH13	Cosmid14E2with *orf4* replaced by *aac(3)IV-oriT* cassette	This work
pDYH14	Cosmid 27D11with *PlyR* replaced by *aac(3)IV-oriT* cassette	This work
pDYH15	Cosmid 14E2with *orf1* replaced by *aac(3)IV-oriT* cassette	This work

Table S3. Primers used in this study

**Primers**	**Sequences**
Probe1F-*plyE*	5´- CATCGACTCGTACGACCTGGAT -3´
Probe1R-*plyE*	5´- CAGTCGAACGCCTCCCCGTCGA -3´
Probe2F-*plyP*	5´- TCCTGGAGAAAGCGAAGTTC -3´
Probe2R-*plyP*	5´- CGTCGTTGAACTCGTCCTGC -3´
PlyH-tgtF	5´-GTGATCCCGCTTTCGTTCGCACAGCGCCGCATGTGGCTCATT CCGGGGATCCGTCGACC -3´
PlyH-tgtR	5´-TCACTCCAGCTCCGCCAACTTGCGCACGATGACGCTCATTG TAGGCTGGAGCTGCTTC -3´
PlyH-cekF	5´- GTGATCCCGCTTTCGTTCGC -3´
PlyH-cekR	5´- TCACTCCAGCTCCGCCAACT -3´
PlyC-tgtF	5´- ATGATCCTTCAGGGAAAGCGGGTGGCCCTGCCCTCGGCGC CCGCATTCCGGGGATCCGTCGACC -3´
PlyC-tgtR	5´- CTACGCCGTGGTGCCGCTGTCGGCGTACCGGTTCCGGTAC GCCTCTGTAGGCTGGAGCTGCTTC -3´
PlyC-cekF	5´- GCGAATTCATGATCCTTCAGGGAAAGCGGGTG -3´
PlyC-cekR	5´- GGTCATCAAGCTTCTACGCCGTGGTGCCGCTGT -3´
PlyD-tgtF	5´-GTGATGATTGCCGAAAGGGTGCGCACCATCTGGTGCCGGGA ACTCATTCCGGGGATCCGTCGACC -3´
PlyD-tgtR	5´- CTACTGGGTCGTCGCACCCAGGGATTCGATGTACGCGGAAA TCGATGTAGGCTGGAGCTGCTTC -3´
PlyD-cekF	5´- GGCTCCACATATGGTGATGATTGCCGAAAGGGTGCGC -3´
PlyD-cekR	5´- GCGGATCCCTACTGGGTCGTCGCACCCAGGGA -3´
PlyQ-tgtF	5´- GTGTCGGAGCGACAAGCCGCGTCGTCGAGTACGCCGGGGT CCCTGATTCCGGGGATCCGTCGACC -3´
PlyQ-tgtR	5´- TCACGGCCGGGCCCTCCCCTGCTCGATCAGCTCGGCGAC CCGGGCTGTAGGCTGGAGCTGCTTC -3´
PlyQ-cekF	5´- GGCTCCACATATGGTGTCGGAGCGACAAGCCGCGTCG -3´
PlyQ-cekR	5´- GCGGATCCTCACGGCCGGGCCCTCCCCTGCTC -3´
PlyI-tgtF	5´- TTGGTCGGGGTGACAACACCCGGCAAGTCCGCGGCGGACC GGGAGATTCCGGGGATCCGTCGACC -3´

PlyI-tgtR	5´- CTAACGATCGCGGGCGGATTCCGCCTCGATCACCGCCAGCA GCTCTGTAGGCTGGAGCTGCTTC -3´
PlyI-cekF	5´- GGCTCCACATATGTTGGTCGGGGTGACAACACCCGGC -3´
PlyI-cekR	5´- GCGGATCCCTAACGATCGCGGGCGGATTCCGC -3´
PlyS-tgtF	5´- ATGACCTCCGGCTCCACCCTCGCCCCGTCGCCATGGTTCGT CCGGATTCCGGGGATCCGTCGACC -3´
PlyS-tgtR	5´- TCACCGCCAAGAGGTCAACAGCTTCAGCAGCTCGTCCGGC GCGTTTGTAGGCTGGAGCTGCTTC -3´
PlyS-cekF	5´- GGCTCCACATATGACCTCCGGCTCCACCCTCGCC -3´
PlyS-cekR	5´- GCGGATCCTCACCGCCAAGAGGTCAACAGCTT -3´
PlyY-tgtF	5´-ATGGCGAGTCCGATGGAAGAGCAGTGGCTGCGCCGCTTCCG TGATATTCCGGGGATCCGTCGACC -3´
PlyY-tgtR	5´- TCAGGCGCCCCCTGCGGTGAGGTGACCCAGTACGTTGCTC AGGTCTGTAGGCTGGAGCTGCTTC -3´
PlyY-cekF	5´- GGCTCCACATATGGCGAGTCCGATGGAAGAGCAG -3´
PlyY-cekR	5´- GCGGATCCTCAGGCGCCCCCTGCGGTGAGGTG -3´
PlyE-tgtF	5´-ATGTACGACGTCATTGTCATAGGTGCCCGTTGTGCGGGTTCG CCGATTCCGGGGATCCGTCGACC -3´
PlyE-tgtR	5´-TCAGTTCAGGTCGAGCAACGTCAAGAGTTCGTCGTTGTAGAA GTCTGTAGGCTGGAGCTGCTTC -3´
PlyE-cekF	5´- GCGAATTCATGTACGACGTCATTGTCATAGGT -3´
PlyE-cekR	5´- GGTCATCAAGCTTTCAGTTCAGGTCGAGCAACGTC -3´
PlyM-tgtF	5´-ATGGCTGAGCCGATCGTTACCCTGACGAACCGAGAATTCACG GACATTCCGGGGATCCGTCGACC -3´
PlyM-tgtR	5´-TCACAGCCGCACCGGAAGCGCGTCGAGCAGTCGGGCGCCGC CGGGTGTAGGCTGGAGCTGCTTC -3´
PlyM-cekF	5´- GGTCAGAGCTCATGGCTGAGCCGATCGTTACCCTG -3´
PlyM-cekR	5´- GGTCATCAAGCTTTCACAGCCGCACCGGAAGCGCG -3´
Orf11-tgtF	5´-ATGCCTGTTTTCGATACACCTGAGCCGATCCACGTCACGCTCG ACATTCCGGGGATCCGTCGACC -3´
Orf11-tgtR	5´-TCAGCTGCGGTGGATCGTGATGTCTCCGTAGGACGTCCGCGC CCGTGTAGGCTGGAGCTGCTTC -3´

Orf11-cekF	5´- ATGCCTGTTTTCGATACACCTG -3´
Orf11-cekR	5´- TCAGCTGCGGTGGATCGTGATG -3´
PlyN-tgtF	5´-ATGGCCATACAACCGGTACGGACGCGATTGAGTATTGCCGATT TAATTCCGGGGATCCGTCGACC -3´
PlyN-tgtR	5´-TCAGGTACGGGCGGCCTCGGCCGGCCACTTTCCGCGCTGATC CCGTGTAGGCTGGAGCTGCTTC -3´
PlyN-cekF	5´- GCGAATTCATGGCCATACAACCGGTACGGA -3´
PlyN-cekR	5´- GGTCATCAAGCTTTCAGGTACGGGCGGCCTCGGCC -3´
PlyP-tgtF	5´-GTGCTGCCCATCGACTCGTACGACCTGGATTCCGAGCTGTCCG TCATTCCGGGGATCCGTCGACC -3´
PlyP-tgtR	5´-TCACCAGTCGAACGCCTCCCCGTCGACCCGCTTCTCGATGCAG TATGTAGGCTGGAGCTGCTTC -3´
PlyP-cekF	5´- GGCTCCACATATGGTGCTGCCCATCGACTCGTACG -3´
PlyP-cekR	5´- GCGGATCCTCACCAGTCGAACGCCTCCCCG -3´
Orf4-tgtF	5´-TTGCCGATCGCCAAGGATCTCGTTGGTTGGATGTGGTCGCGCG AAATTCCGGGGATCCGTCGACC -3´
Orf4-tgtR	5´-TCACCCTCGCCGGGCGGGCCCGAACCAGGTGCCGAGGGCGG TCCGTGTAGGCTGGAGCTGCTTC -3´
Orf4-cekF	5´- TTGCCGATCGCCAAGGATCTCG -3´
Orf4-cekR	5´- TCACCCTCGCCGGGCGGGCCCG -3´
PlyR-tgtF	5´-GTGAGGTTGACGCCGGGTGCCGGGGGCGACGTCGACCTGGA CCGCATTCCGGGGATCCGTCGACC -3´
PlyR-tgtR	5´-TCATGGCGTCTTTCGTTTCTGGGCGACGATGGGGAGGTGGGTC ATTGTAGGCTGGAGCTGCTTC -3´
PlyR-cekF	5´- GCGAATTCGTGAGGTTGACGCCGGGTGCCG -3´
PlyR-cekR	5´- GGTCATCAAGCTTTCATGGCGTCTTTCGTTTCTGG -3´
Orf1-tgtF	5´-GTGCTCGCCGTAAGTATTTCGGCAGCGAATATCCGGATTGTCT ACATTCCGGGGATCCGTCGACC -3´
Orf1-tgtR	5´-TCAGCCATCGGTGCCTCCGTGCGCGCGGGCGAGGCTGACCTC GGCTGTAGGCTGGAGCTGCTTC -3´
Orf1-cekF	5´- GTGCTCGCCGTAAGTATTTCGG -3´
Orf1-cekR	5´- TCAGCCATCGGTGCCTCCGTGC -3´

F stands for forward primer, and R stands for reverse primer. Cek indicates the primers used for diagnostic PCR.

Table S4. The substrate specificity codons of seven A domains.

A domain	Conserved motif	Proposed substrate
PlyC-A	DLRHLGQDVK	unknown
PlyF-A1	DVFSVASYAK	Piperazic acid
PlyF-A2	DVPDEALVEK	N-hydroxyl-D-alanine
PlyG-A1	DVFSIAAYAK	Piperazic acid
PlyG-A2	DVFCNSSYAK	3-hydroxyl-3-methyl-proline
PlyH-A	DAPFEALVEX	N-hydroxyl-L-valine
PlyX-A	DTLWWGGGFR	3-hydroxyl-leucine

2. Supplementary Figures

Figure S1. Alignment of four KS domains of the *ply* gene cluster

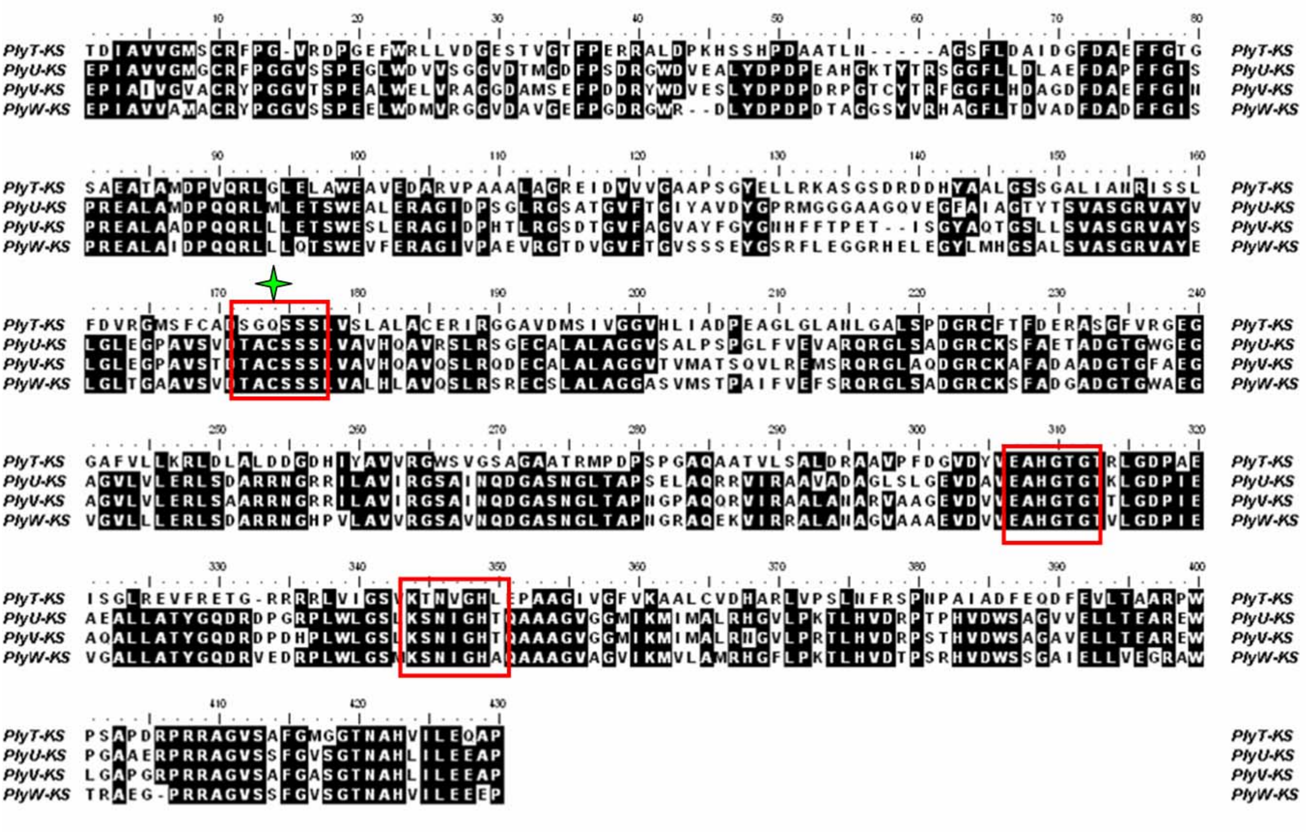

Figure S1. Alignment of four KS domains of the *ply* cluster. The boxed parts indicate the conserved mitifs for KS domains. The star indicates the replacement of the transthioesterification site Cys(C) of PlyT-KS with Gln(Q)

Figure S2   Alignment of four AT domains

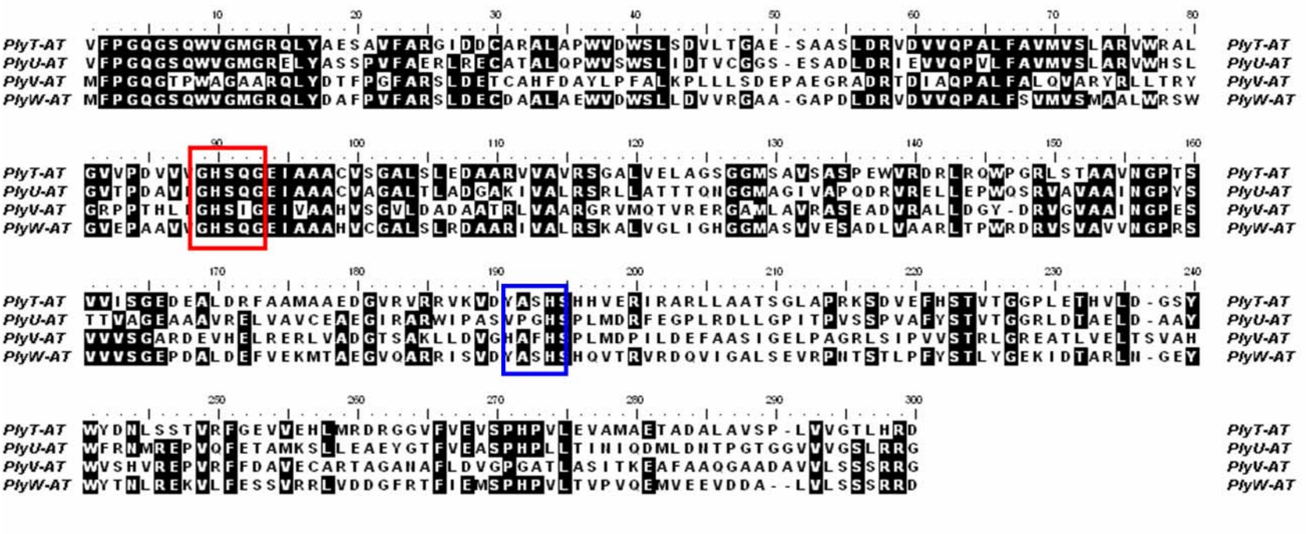

Figure S2   Alignment of four AT domains of the *ply* cluster. The red box indicates the conserved active motif and the blue box indicates the motif for the substrate specificity.

Figure S3 Alignment of DH domains

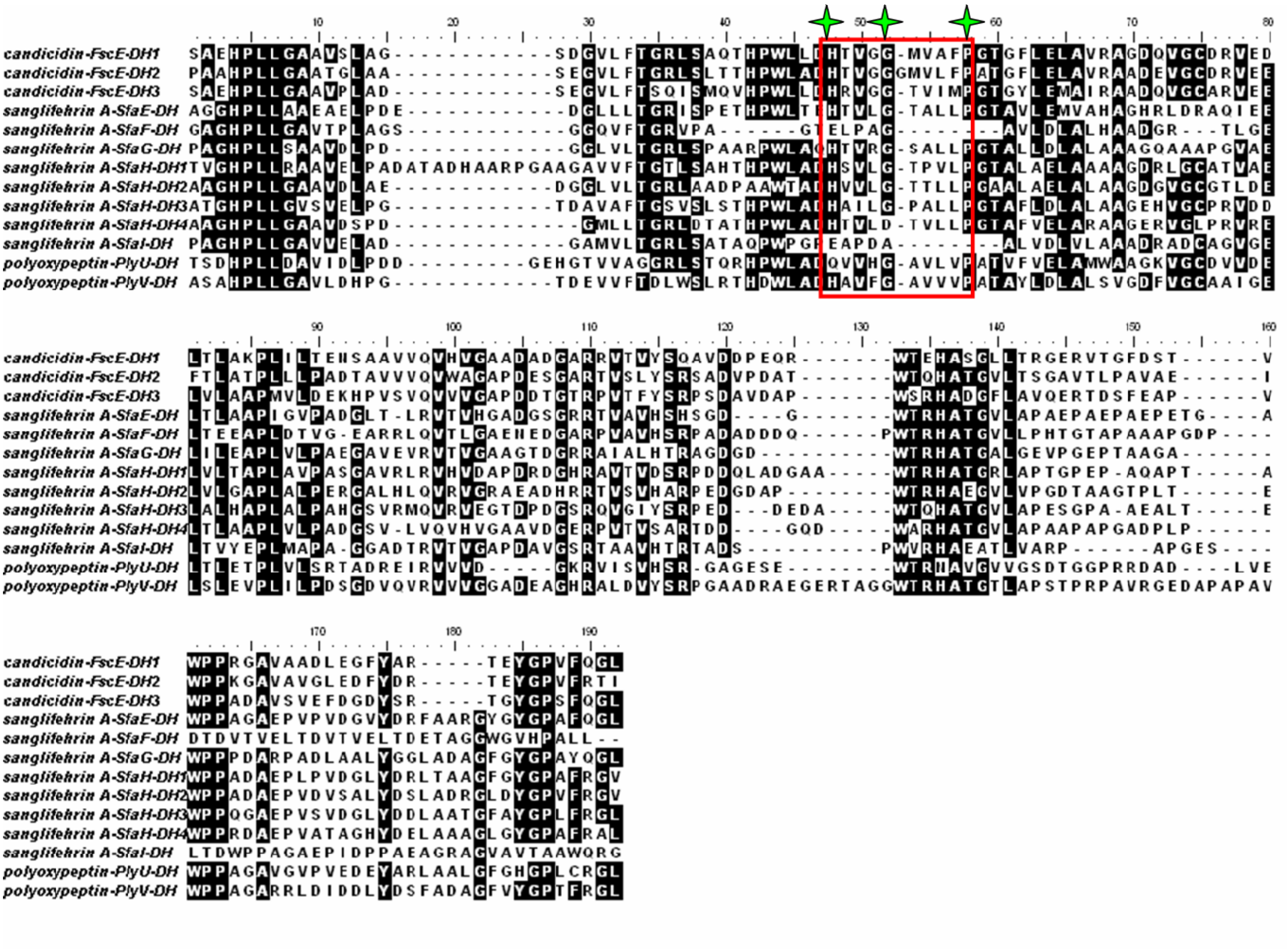

Figure S3 Alignment of DH domains. The box indicates the conserved motif of DH domains and the stars indicate the active sites. DH amino acid sequence from *Streptomyces* sp. FR-008 (Accession no.AY310323): Candicidin-FscE-DH1, Candicidin-FscE-DH2(inactive) and Candicidin-FscE-DH3; DH amino acid sequence from *Streptomyces flaveolus* strain DSM 9954 (Accession no.FJ809786): Sanglifehrin A-SfaE-DH, Sanglifehrin A-SfaF-DH(inactive), Sanglifehrin A-SfaG-DH, Sanglifehrin A-SfaH-DH1, Sanglifehrin A-SfaH-DH2, Sanglifehrin A-SfaH-DH3, Sanglifehrin A-SfaH-DH4 and Sanglifehrin A-SfaI-DH(inactive).

Figure S4 Alignment of A domains activating piperazic acid

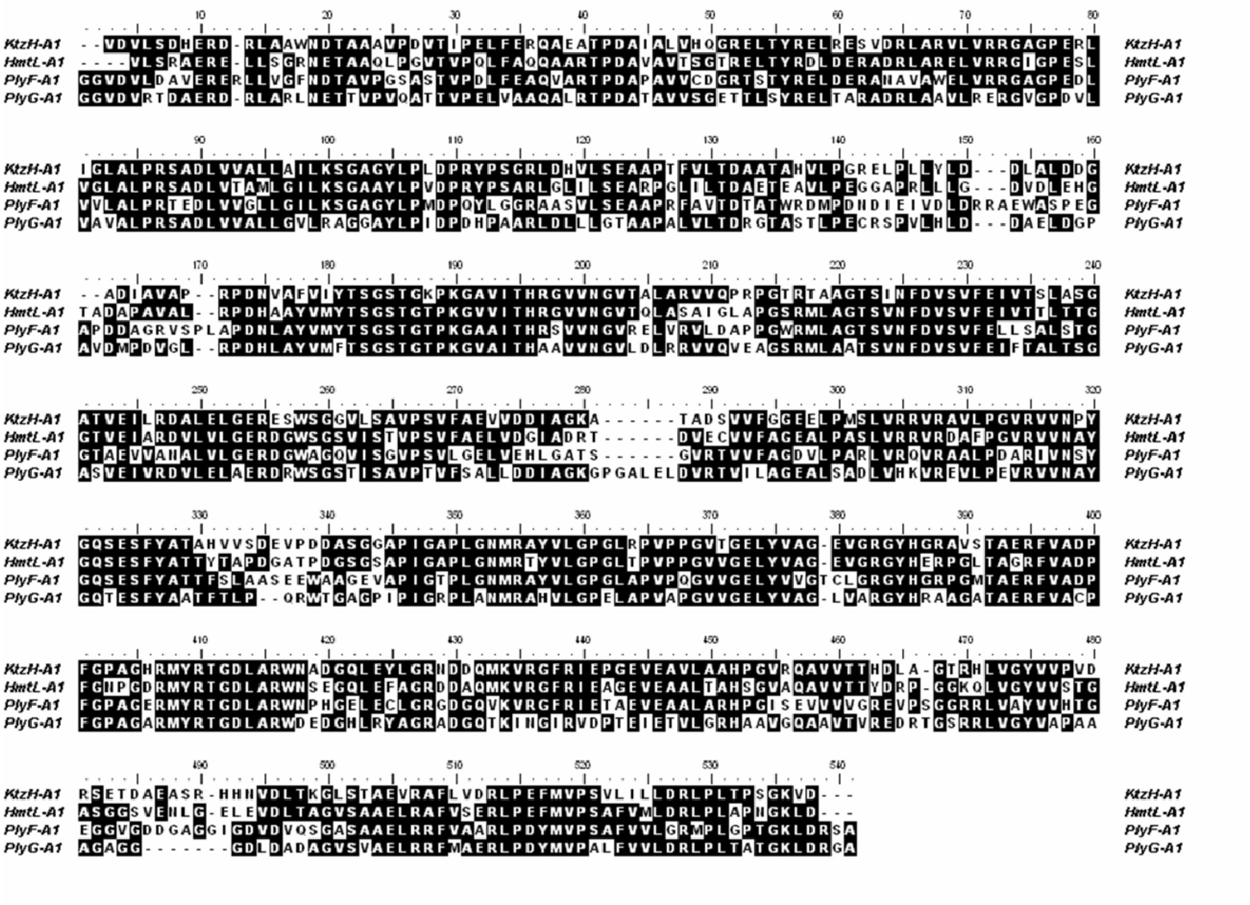

Figure S4 Alignment of A domains activating piperazic acid. KtzH-A1: the amino acid sequence of A domain of KtzH from *Kutzneria* sp.744 (Accession no.EU074211); HmtL-A1: the amino acid sequence of A domain of HmtL from *Streptomyces himastatinicus* ATCC 53653 (Accession no.FR823394).

Figure S5 Alignment of PlyF-A2 and PlyH-A

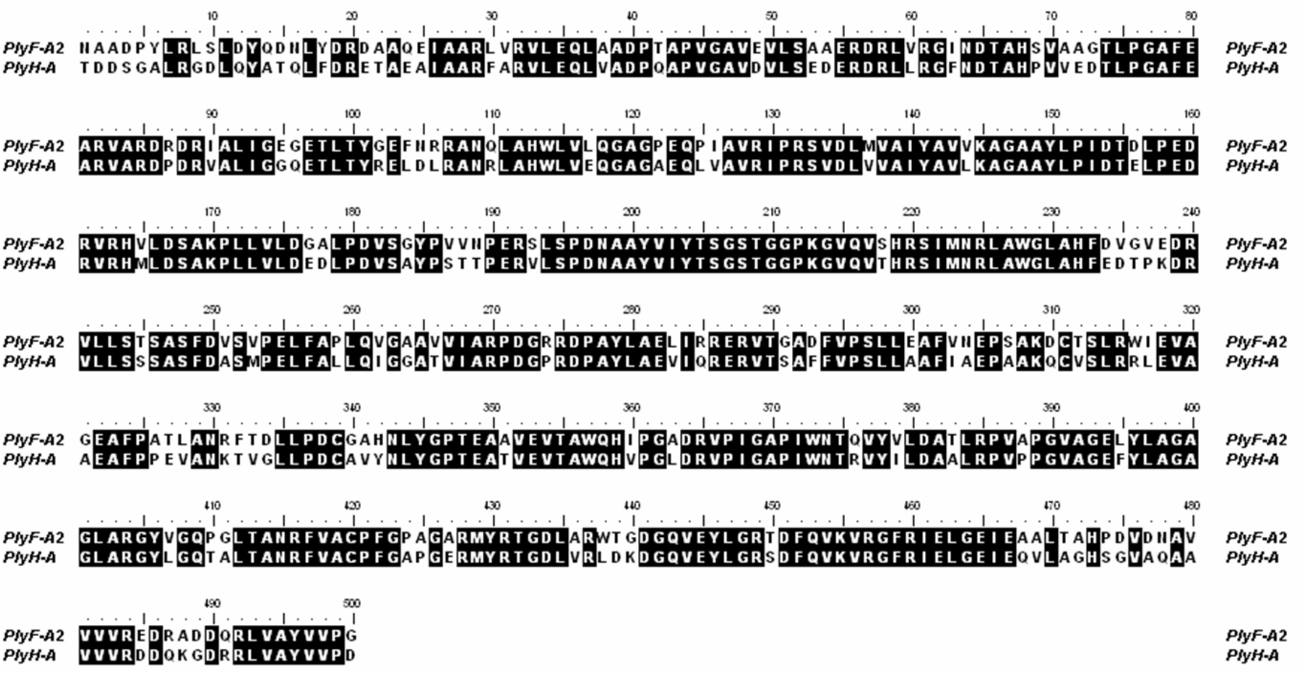

Figure S5 Alignment of PlyF-A2 and PlyH-A. They share 76% identity and 83% similarity

Figure S6 Alignment of Type I and Type II TE domains

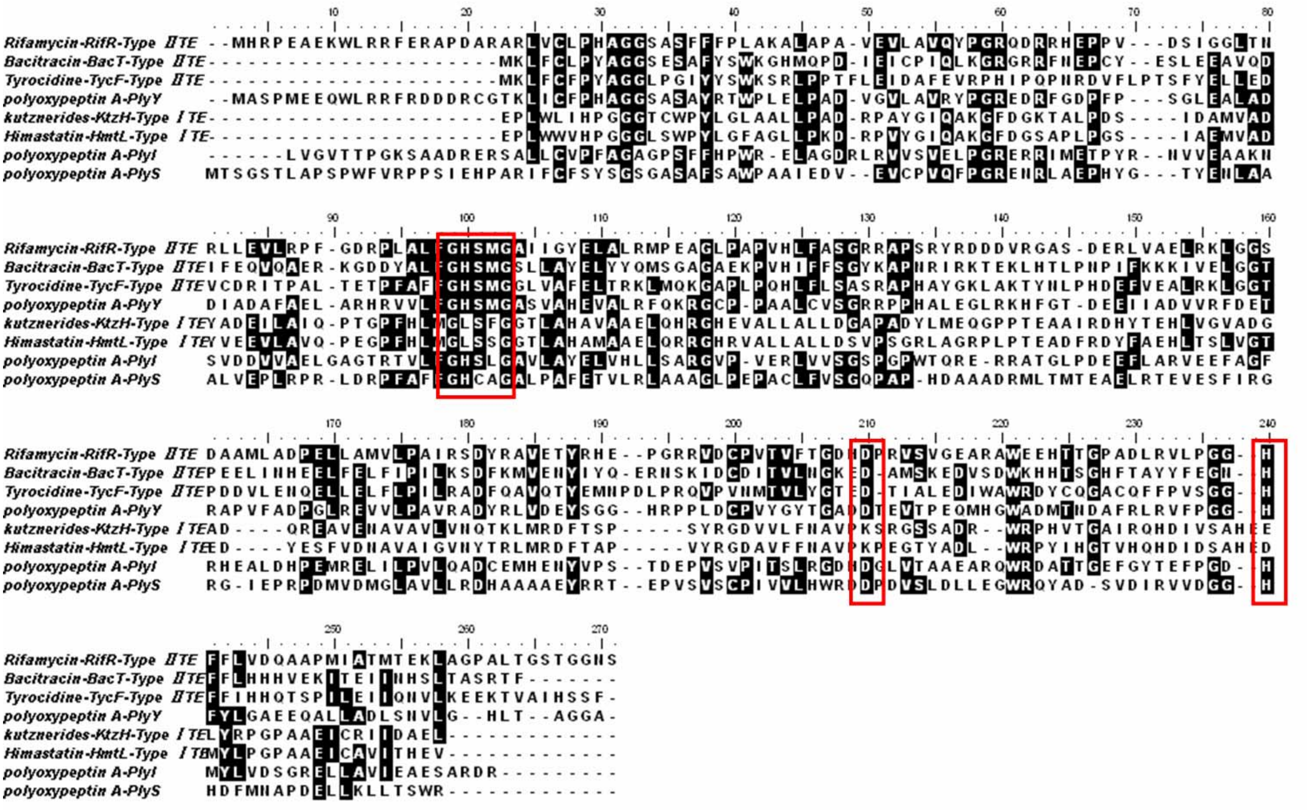

Figure S6 Alignment of Type I and Type II TE domains. The boxes indicate the conserved motif and active sites. Rifamycin-RifR-Type II TE: the amino acid sequence of type II TE RifR from *Amycolatopsis mediterranei* S699 (Accession no.AF040570); Bacitracin-BacT-Type II TE: the amino acid sequence of type II TE BacT from *Bacillus licheniformis* (Accession no.AF007865); Tyrocidine-TycF-Type IITE: the amino acid sequence of type II TE TycF from *Brevibacillus brevis* (Accession no.AF004835); Kutznerides-KtzH-Type I TE: the amino acid sequence of type I TE in KtzH from *Kutzneria* sp.744 (Accession no.EU074211); Himastatin-HmtL-Type I TE: the amino acid sequence of type I TE in HmtL from *Streptomyces himastatinicus* ATCC 53653 (Accession no.FR823394).

3. Supplementary Schemes

Scheme S1. Construction and the genotype verification of the Δ*plyH* mutant DYH1

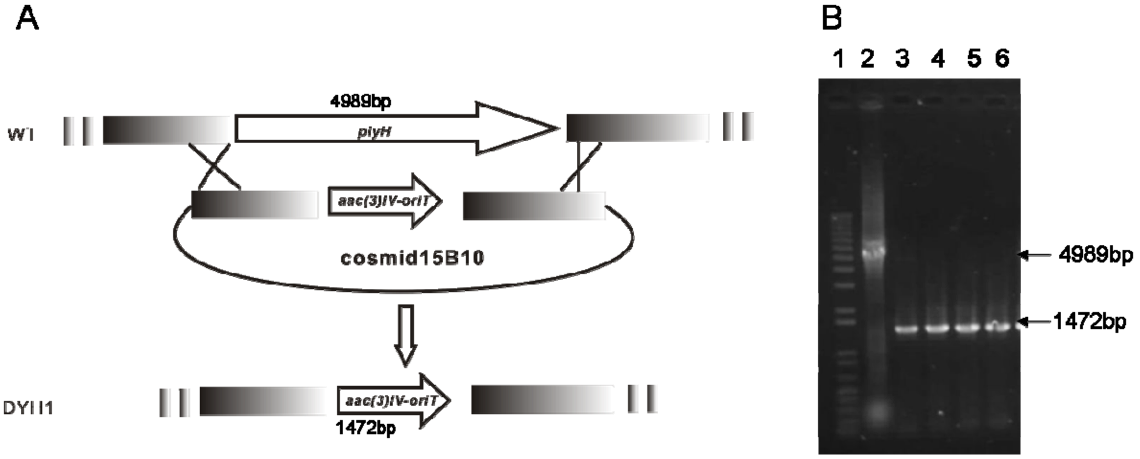

Scheme S1. Construction and the genotype verification of the Δ*plyH* mutant DYH1. A. Construction of the Δ*plyH* mutant DYH1 by replacement of *plyH* with apramycin-resistant/oriT cassette (*aac(3)IV-oriT*); B. Confirmation of the inactivation of *plyH* by insertion of apramycin-resistant/oriT cassette (*aac(3)IV-oriT*) by PCR using the primers listed in Table S3; Lane 1: molecular weight marker; Lane 2: PCR analysis of wild-type strain; Lane 3-6: PCR analysis of DYH1.

Scheme S2. Construction and the genotype verification of the Δ*plyN* mutant DYH11

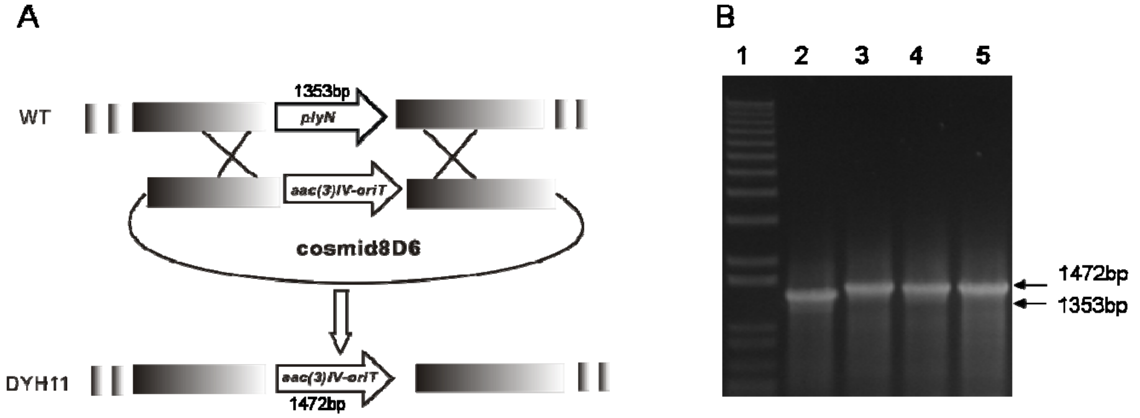

Scheme S2. Construction and the genotype verification of the Δ*plyN* mutant DYH11. A. Construction of the Δ*plyN* mutant DYH11 by replacement of *plyN* with apramycin-resistant/oriT cassette (*aac(3)IV-oriT*); B. Confirmation of the inactivation of *plyN* by insertion of apramycin-resistant/oriT cassette (*aac(3)IV-oriT*) by PCR using the primers listed in Table S3; Lane 1: molecular weight marker; Lane 2: PCR analysis of wild-type strain; Lane 3-5: PCR analysis of DYH11.

Scheme S3. Construction and the genotype verification of the Δ*plyC* mutant DYH2

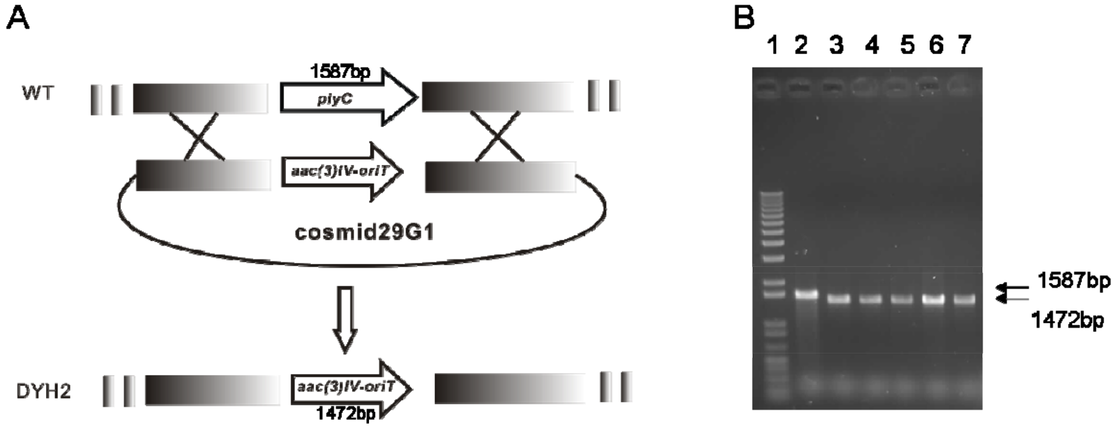

Scheme S3. Construction and the genotype verification of the Δ*plyC* mutant DYH2. A. Construction of the Δ*plyC* mutant DYH11 by replacement of *plyC* with apramycin-resistant/oriT cassette (*aac(3)IV-oriT*); B. Confirmation of the inactivation of *plC* by insertion of apramycin-resistant/oriT cassette (*aac(3)IV-oriT*) by PCR using the primers listed in Table S3; Lane 1: molecular weight marker; Lane 2: PCR analysis of wild-type strain; Lane 3-7: PCR analysis of DYH2.

Scheme S4. Construction and the genotype verification of the Δ*plyD* mutant DYH3

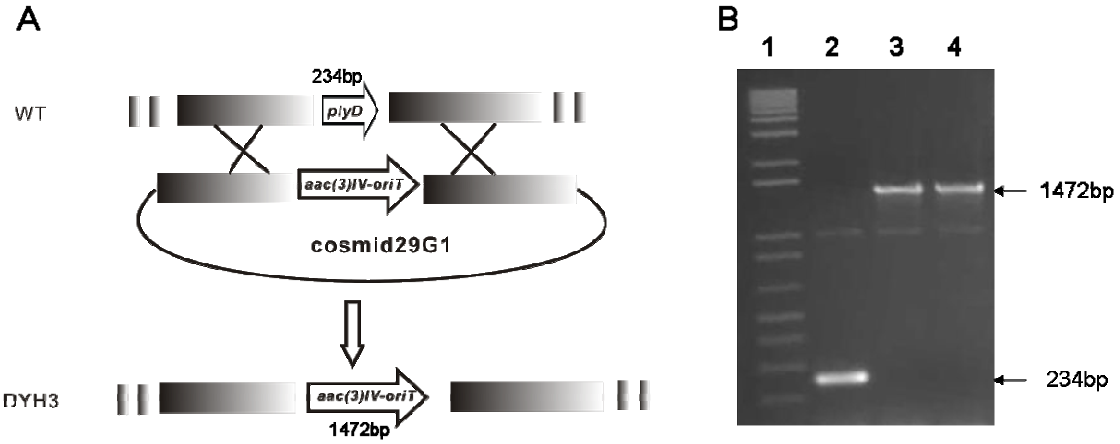

Scheme S4 Construction and the genotype verification of the Δ*plyD* mutant DYH3. A. Construction of the Δ*plyD* mutant DYH11 by replacement of *plyD* with apramycin-resistant/oriT cassette (*aac(3)IV-oriT*); B. Confirmation of the inactivation of *plyD* by insertion of apramycin-resistant/oriT cassette (*aac(3)IV-oriT*) by PCR using the primers listed in Table S3; Lane 1: molecular weight marker; Lane 2: PCR analysis of wild-type strain; Lane 3-4: PCR analysis of DYH3

Scheme S5. Construction and the genotype verification of the Δ*plyQ* mutant DYH4

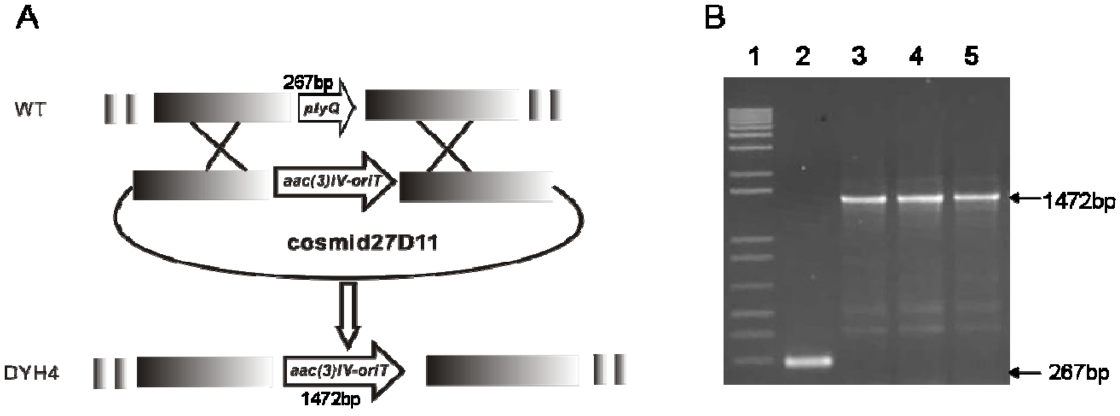

Scheme S5. Construction and the genotype verification of the Δ*plyQ* mutant DYH4. A. Construction of the Δ*plyO* mutant DYH11 by replacement of *plyO* with apramycin-resistant/oriT cassette (*aac(3)IV-oriT*); B. Confirmation of the inactivation of *plyO* by insertion of apramycin-resistant/oriT cassette (*aac(3)IV-oriT*) by PCR using the primers listed in Table S3; Lane 1: molecular weight marker; Lane 2: PCR analysis of wild-type strain; Lane 3-5: PCR analysis of DYH4.

Scheme S6. Construction and the genotype verification of the Δ*plyI* mutant DYH5

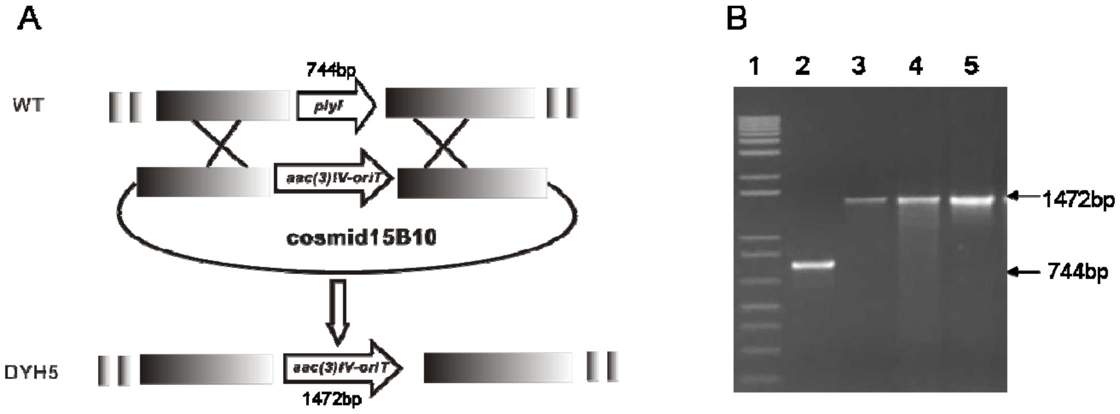

Scheme S6. Construction and the genotype verification of the Δ*plyI* mutant DYH5. A. Construction of the Δ*plyI* mutant DYH11 by replacement of *plyI* with apramycin-resistant/oriT cassette (*aac(3)IV-oriT*); B. Confirmation of the inactivation of *plyI* by insertion of apramycin-resistant/oriT cassette (*aac(3)IV-oriT*) by PCR using the primers listed in Table S3; Lane 1: molecular weight marker; Lane 2: PCR analysis of wild-type strain; Lane 3-5: PCR analysis of DYH5.

Scheme S7. Construction and the genotype verification of the Δ*plyS* mutant DYH6

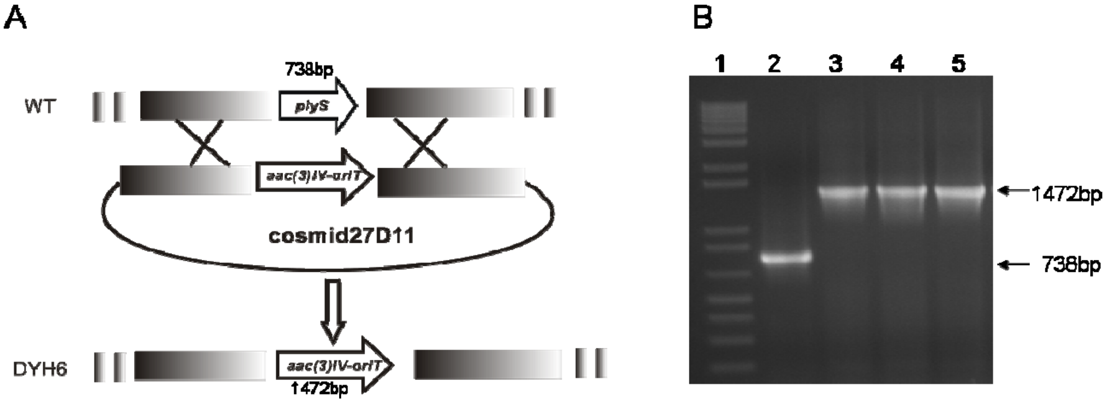

Scheme S7. Construction and the genotype verification of the Δ*plyS* mutant DYH6. A. Construction of the Δ*plyS* mutant DYH11 by replacement of *plyS* with apramycin-resistant/oriT cassette (*aac(3)IV-oriT*); B. Confirmation of the inactivation of *plyS* by insertion of apramycin-resistant/oriT cassette (*aac(3)IV-oriT*) by PCR using the primers listed in Table S3; Lane 1: molecular weight marker; Lane 2: PCR analysis of wild-type strain; Lane 3-5: PCR analysis of DYH6.

Scheme S8. Construction and the genotype verification of the Δ*plyY* mutant DYH7

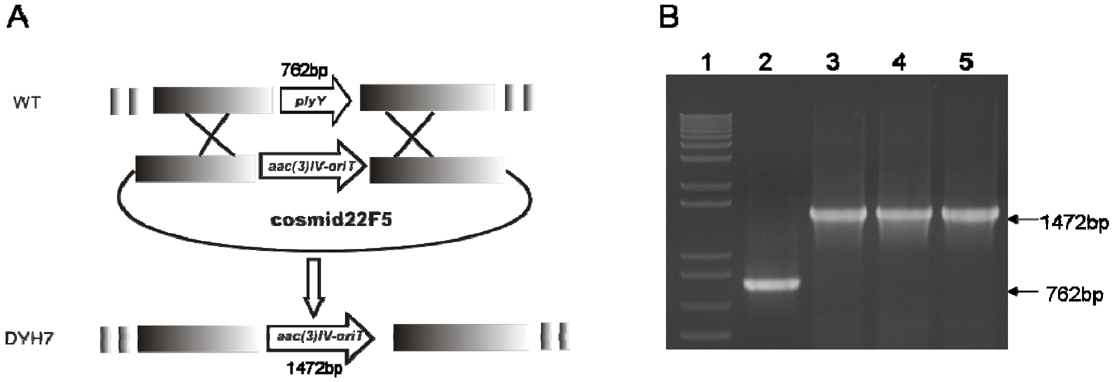

Scheme S8. Construction and the genotype verification of the Δ*plyY* mutant DYH7. A. Construction of the Δ*plyY* mutant DYH11 by replacement of *plyY* with apramycin-resistant/oriT cassette (*aac(3)IV-oriT*); B. Confirmation of the inactivation of *plyY* by insertion of apramycin-resistant/oriT cassette (*aac(3)IV-oriT*) by PCR using the primers listed in Table S3;Lane 1: molecular weight marker; Lane 2: PCR analysis of wild-type strain; Lane 3-5: PCR analysis of DYH7.

Reference:

1. Umezawa K, Nakazawa K, Ikeda Y, Naganawa H, Kondo S.1999. Polyoxypeptins A and B Produced by Streptomyces: Apoptosis-Inducing Cyclic Depsipeptides Containing the Novel Amino Acid (2S, 3R)-3-Hydroxy-3-methylproline. J Org Chem. 64(9):3034-3038.
2. Simon, R., U. Priefer, and A. Pu¨hler. 1983. A broad host range mobilization system for *in vivo* genetic engineering: transposon mutagenesis in gram negative bacteria. Biotechnology. 1**:**784–791.
